# Mechanotransduction-induced interplay between phospholamban and yes-activated protein induces smooth muscle cell hypertrophy

**DOI:** 10.1016/j.mucimm.2024.02.007

**Published:** 2024-02-27

**Authors:** Renee Rawson, Loan Duong, Eugene Tkachenko, Austin W.T. Chiang, Kevin Okamoto, Ranjan Dohil, Nathan E. Lewis, Richard Kurten, Edsel M. Abud, Seema S. Aceves

**Affiliations:** 1 Department of Pediatrics, University of California, San Diego, California.; 2 Division of Allergy Immunology, University of California, San Diego, California.; 3 Department of Bioengineering, University of California, San Diego, California.; 4 Division of Gastroenterology, University of California, San Diego, California.; 5 Department of Physiology and Biophysics, University of Arkansas for Medical Sciences, Arkansas Children’s Hospital Research Institute, Little Rock, Arkansas.; 6 XXX, Rady Children’s Hospital, San Diego, California.; 7 Department of Medicine, University of California, San Diego, California.; 8 XXX, Scripps Research Translational Institute, Scripps Research, San Diego, California.

## Abstract

The gastrointestinal system is a hollow organ affected by fibrostenotic diseases that cause volumetric compromise of the lumen via smooth muscle hypertrophy and fibrosis. Many of the driving mechanisms remain unclear. Yes-associated protein-1 (YAP) is a critical mechanosensory transcriptional regulator that mediates cell hypertrophy in response to elevated extracellular rigidity. In the type 2 inflammatory disorder, eosinophilic esophagitis (EoE), phospholamban (PLN) can induce smooth muscle cell hypertrophy. We used EoE as a disease model for understanding a mechanistic pathway in which PLN and YAP interact in response to rigid extracellular substrate to induce smooth muscle cell hypertrophy. PLN-induced YAP nuclear sequestration in a feed-forward loop caused increased cell size in response to a rigid substrate. This mechanism of rigidity sensing may have previously unappreciated clinical implications for PLN-expressing hollow systems such as the esophagus and heart.

## INTRODUCTION

Chronic diseases of hollow organs can lead to lumen stenosis that impacts patient morbidity and mortality^1–4^. These diseases include those of the heart, such as cardiomyopathy, the lung, such as asthma, and the esophagus, such as eosinophilic esophagitis (EoE)^5–7^. In the esophagus, EoE is an increasingly prevalent chronic type 2 inflammatory disorder that has become a common cause of esophageal food impaction and strictures^8–10^. Progressive EoE causes histologic, endoscopic, and functional tissue rigidity with decreased esophageal lumen cross-sectional area^3,8,11,12^. The pathogenesis of tissue rigidity and lumen stenosis in EoE is driven by muscular hypertrophy and fibrosis, as evident in ultrasound and histological studies^5,8^. We have previously demonstrated that phospholamban (PLN) expression in smooth muscle cells (SMCs) of the esophagus is increased in chronic EoE^12–14^. We have also demonstrated that PLN can be induced in esophageal SMCs cultured on rigid substrates, suggesting PLN expression is driven by mechanotransducive signaling^12–14^. While PLN mutations have been reported to cause cardiomyopathy^15^, a subset of which can be hypertrophic^15^, its role in esophageal muscle hypertrophy and its contribution to esophageal disease is understudied^15^.

Mechanotransduction is a complex process by which cells sense extracellular environmental rigidity to alter their molecular functions. Altered cellular structure including perturbed dynamics of the actin cytoskeleton and resultant changes in the nuclear envelope and pore cause ubiquitination, phosphorylation, and nuclear-cytoplasmic shuttling of transcriptional regulators such as co-activator, yes-associated protein-1 (YAP)^16^. Via nuclear shuttling, YAP alters transcriptional cascades and cellular function resulting in processes that are pivotal for human development, including the esophagus^17^, and in disease. In cardiac and skeletal muscle, YAP is known to induce cell hypertrophy^18^. In pancreatic cancer, YAP expression along rigid foci is associated with metastases^19^. Thus, the role of YAP in cellular size and function has been clearly delineated. However, whether YAP and PLN can mechanistically interact with one another in a rigidity-sensing pathway is not clear. Here, we utilized normal primary SMCs from human esophagi and EoE^12,14^ as a model system to understand the mechanism that links tissue rigidity to PLN induction and SMC hypertrophy.

## RESULTS AND DISCUSSION

### PLN and SMC hypertrophy

**Fig. 1:**
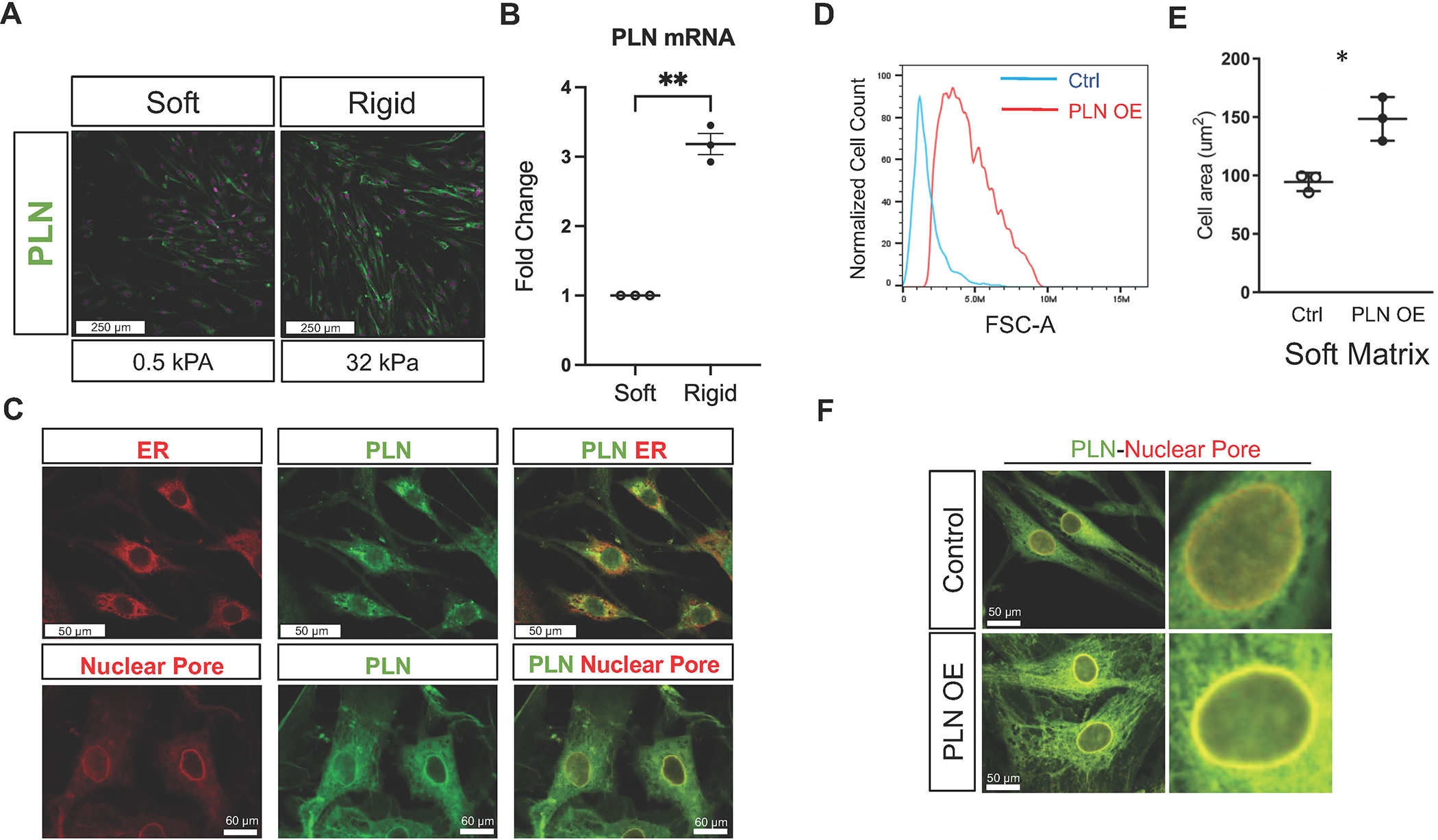
Phospholamban localization and response to rigid substrate. (A) Representative image of primary esophageal smooth muscle cells cultured on soft (0.5 kPa) and rigid (32 kPa) matrix. PLN immunofluorescence (green) and nuclei (DAPI, magenta) imaged at the same gain and magnification for each condition using 20x objective. <scale bar = 250 μm>. Repeated using four different donors. (B) PLN mRNA expression (fold change) of cells cultured on soft versus rigid substrate. Dots = different individuals. **p < 0.0048. (C) Representative images of co-localization of PLN (green) and endoplasmic reticulum (Calnexin, ER, red) or nuclear pore (Nuclear Pore Complex, red) (overlap, yellow) in primary human esophageal smooth muscle cells. <scale bar top = 50 μm, bottom = 60um>. (D) Representative histogram of baseline size on flow cytometry using stable transgenic PLN overexpression (PLN-OE) or control GFP expression vector in SMCs. (E) Representative experiment of esophageal SMCs cultured on soft (0.2 or 0.5kPa) substrate for 15 days, transduced with PLN or GFP (control) expression virus on day 10 with cell area quantified on day 15. Repeated with three different donors. Dots: 3–5 high power fields quantified at 20x light microscopy, Lines: Mean ± SD, *p < 0.05; Unpaired t test. (F) Representative image of SMCs with stable transgenic PLN (green) co-localization with nuclear pore proteins (red, overlap = yellow). <scale bar = 50 μm>. DAPI = 4’,6-diamidino-2-phenylindole; ER = endoplasmic reticular; GFP = green fluorescent protein; kPA = kilopascals; mRNA = messenger ribonucleic acid; PLN = phospholamban; PLN-OE = phospholamban overexpression; SD = standard deviation; SMC = smooth muscle cell.

Full-thickness muscle hypertrophy occurs in EoE^2^, and our prior data demonstrated PLN expression in the esophageal smooth muscle in active EoE patient biopsies^13^. Because of these *in vivo* findings, we elected to use primary esophageal SMCs to study PLN-based mechanisms of hypertrophy. Organ donor-derived primary esophageal SMCs^12^ were cultured on collagen-coated bioengineered substrates of rigid and soft Young’s moduli [kilopascals (kPa)]^14^. SMCs cultured on rigid substrate were larger and PLN immunofluorescence as well as messenger ribonucleic acid (mRNA) expression were increased (Figs. 1A and 1B). PLN co-localized with markers of the endoplasmic reticulum (Calnexin) and nuclear pore (Nuclear Pore Complex) (Fig. 1C).

We confirmed PLN expression in the nuclear pore and its capacity to induce SMC hypertrophy using SMCs stably transduced with PLN or control [green fluorescent protein (GFP)] expression vectors. Stably transduced PLN SMCs were larger using flow cytometry analysis (Fig. 1D). SMCs expressing PLN were hypertrophic despite culture on soft substrate (Fig. 1E). Like endogenous PLN, transduced PLN localized to the nuclear pore (Fig. 1F), suggesting that it may have effects on transcription or nuclear factors. Together, these findings reveal that SMC size and PLN increases in response to rigid substrate can be recapitulated by transgenic expression of PLN on soft matrix and demonstrate PLN’s capacity to phenocopy the SMC hypertrophy induced by rigid substrate. We next set out to understand the mechanism by which PLN expression leads to SMC hypertrophy in response to rigid substrate.

### YAP is increased in EoE and hypertrophic esophageal SMCs

**Fig. 2:**
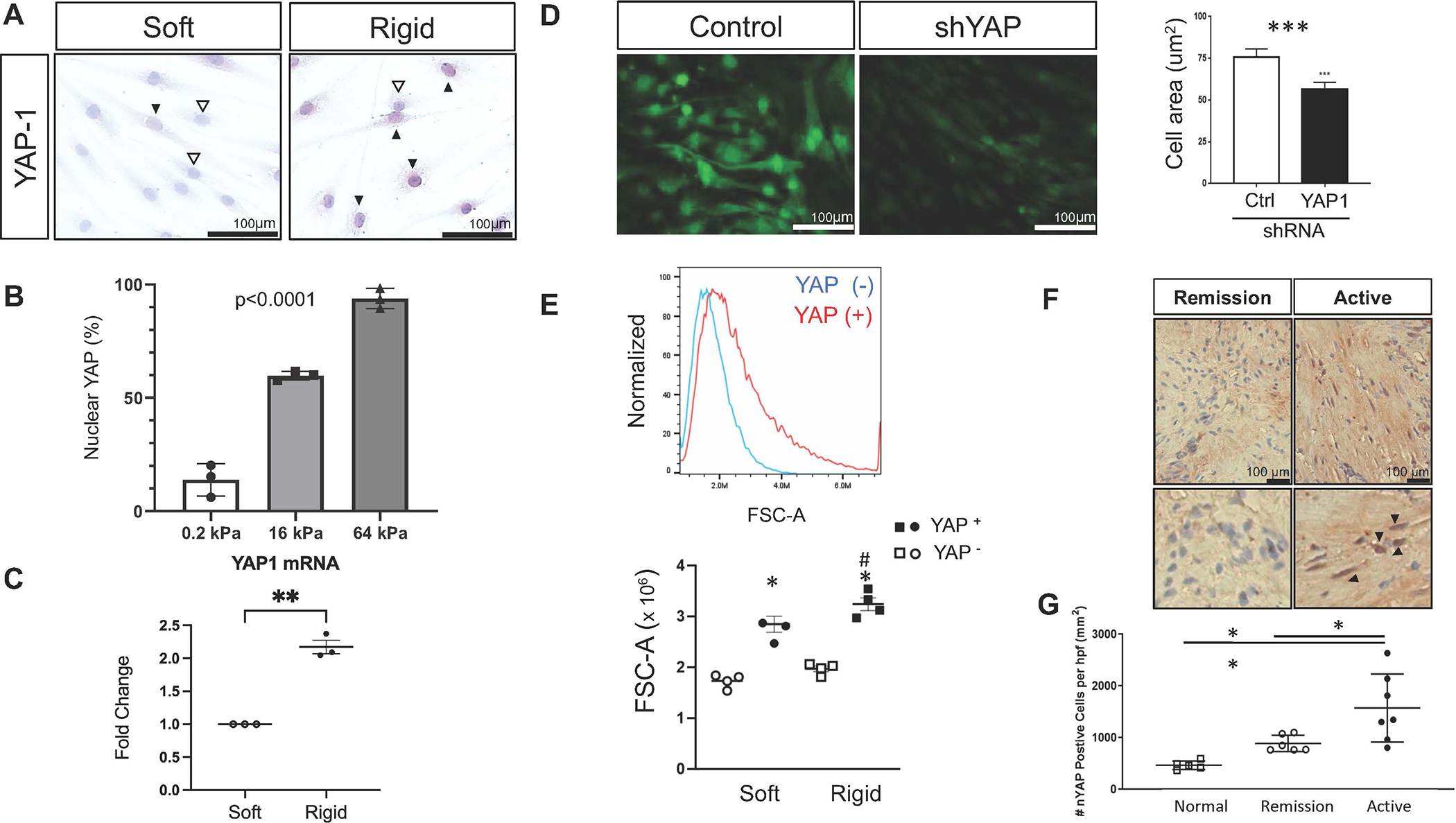
Esophageal smooth muscle expression of yes-associated protein. (A) Representative image of immunostaining for nuclear YAP in esophageal SMCs cultured on soft (0.5–2kPa) or rigid (16–32kPa, 64–96kPa) substrate for 5 days. Repeated using SMCs from six different donors. <scale bar = 100 μm>. (B) Quantification (percent total) of YAP+ cells cultured on increasing rigid substrates. ***p < 0.0001 (C) Representative Fold Change over Soft matrix mRNA expression of YAP in SMCs cultured on soft versus rigid surface. **p < 0.0076, unpaired t-test with Welch’s correction. Repeated using three different donor SMCs. (D) Left: Representative images and quantification of cell surface area in SMCs with YAP silencing using shRNA for YAP versus non-silencing control (GFP; control). <scale bar = 100 μm>. Right: Bar graph of Means ± SD of 3–5 high power fields. Repeated using three different donor SMCs. ***p < 0.005, t-test. <scale bar = 100 μm>. (E) Top: Representative histogram of naturally occurring YAP+ and YAP− SMCs. Bottom: SMC size [forward scatter area (FSC-A)] on soft or rigid substrate using flow cytometry analysis. Dots: different donors, Lines: Mean ± SEM; *p < 0.0001, #: *p* < 0.01. Two-way analysis of variance with Sidak’s multiple comparisons. (F) Representative images of YAP staining in smooth muscle bundles of paraffin-embedded esophageal biopsies from donor esophagus or children with remission or severe active EoE. Arrowheads indicate red YAP+ nuclei. <scale bar = 100 μm>. (G) Quantitation of YAP+ nuclei in the smooth muscle bundles of controls (donor), remission EoE, and severe active EoE biopsies. Dots: different patients, Lines: Mean ± SEM; *: *p* < 0.05, **p < 0.01. One-way analysis of variance with Tukey’s multiple comparisons. FSC-A = forward scatter area A; GFP = green fluorescent protein; kPA = kilopascals; mRNA = messenger ribonucleic acid; SEM = standard error of the mean; shRNA = short hairpin ribonucleic acid; SMC = smooth muscle cell; YAP = yes-associated protein.

Previous studies have demonstrated that muscle cell hypertrophy is driven by YAP nuclear localization in response to rigid cell substrate^4^. We found that nuclear YAP accumulation in esophageal SMCs occurs in response to an increasingly rigid substrate (Figs. 2A and 2B). YAP nuclear accumulation could occur via translocation in response to changes in its phosphorylation state, in response to altered actin cytoskeleton dynamics, and/or via increased gene transcription. We found that rigid substrate induced YAP gene expression in SMCs (Fig. 2C). These data demonstrated that esophageal SMCs have both increased YAP expression and increased YAP nuclear accumulation in response to rigid substrates, suggesting behavior like that observed in other cell types involved in self-renewal, proliferation, and wound healing in health and disease^4^.

Having demonstrated that YAP expression and nuclear localization increase with rigid substrate, we next sought to inhibit YAP expression in esophageal SMCs using shRNA silencing of YAP (shYAP). shYAP SMCs had reduced baseline cell surface area via immunofluorescence analysis (Fig. 2D). In culture, naturally occurring YAP+ cells were larger in size as assessed by flow cytometry (Fig. 2E, top), confirming that YAP expression altered SMC size. Strikingly, naturally occurring YAP^+^ SMCs cultured on rigid substrates had a significant increase in their size by flow cytometry but YAP^−^ SMCs on rigid substrates did not respond with hypertrophy as assessed by flow cytometry (Fig. 2E, bottom). These data support that YAP expression is required for mechanotransduction of rigid substrate signals driving SMC hypertrophy.

Severe EoE is known to be associated with tissue rigidity, and we previously demonstrated that PLN is expressed in the smooth muscle bundles of active EoE patients. We sought to evaluate YAP presence via immunohistochemical study in biopsies from patients with severe fibrostenotic EoE. Patients with severe and stricture-associated EoE, and therefore esophageal rigidity, had significantly more smooth muscle bundles with detectable nuclear YAP as compared to remission EoE and normal biopsies (Figs. 2F and 2G). These data support that YAP drives esophageal SMC hypertrophy via mechanotransduction due to matrix rigidity and demonstrate that patients with a rigid esophagus have smooth muscle bundles that are richer in nuclear YAP expression.

### PLN and YAP interact to mediate mechanotransduction driven smooth muscle hypertrophy

**Fig. 3:**
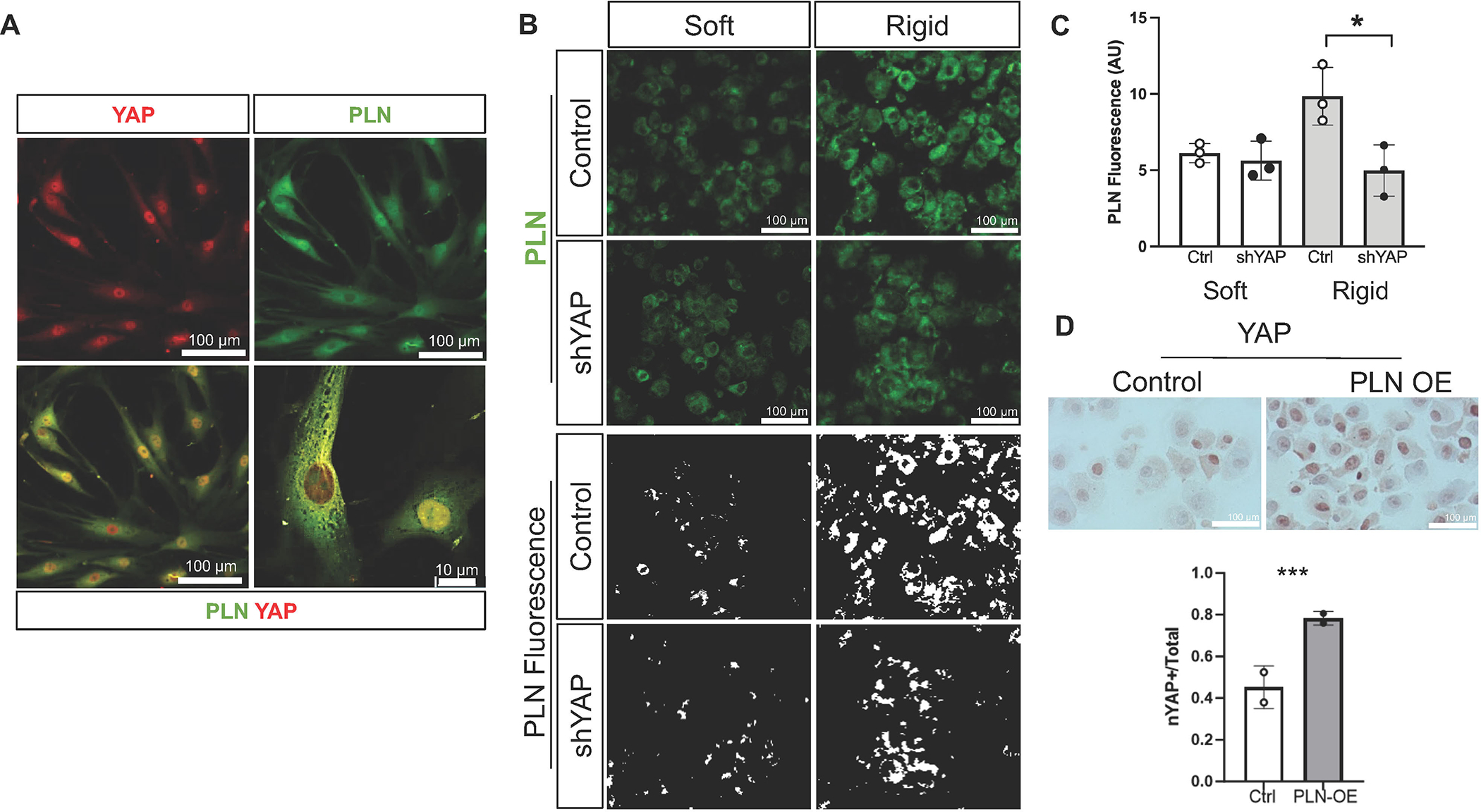
Interaction between phospholamban, yes-associated protein, and rigid substrate. (A) Representative images of YAP (red) and PLN (green) colocalization (yellow) in esophageal SMCs. <scale bar = 100 μm>. (B) Representative images of PLN expression in YAP silenced (shYAP) or non-silencing control (GFP; control) transduced esophageal SMCs cultured on soft (0.5 kPa) or rigid (32 kPa) substrates. Cells were trypsinized, collected, cyto-spun, and stained for PLN (top: green, bottom: inverted fluorescence images in black and white). <scale bar = 100 μm>. (C) Quantification of PLN fluorescence in arbitrary units (AU). Repeated twice using esophageal smooth muscle cell from a single donor. Bars, lines: Mean ± SD of 3–5 high power fields. *: *p* < 0.05 using two-way analysis of variance with Sidak’s multiple comparisons. (D) (Top) Representative images of YAP immunostaining in PLN or control vector (GFP; control) transduced smooth muscle cells cultured on soft substrate (0.5 kPa) substrate for 4 days. <scale bar = 100 μm>. Cells were trypsinized, collected, cyto-spun, and stained for YAP. (Bottom) Quantified nuclear YAP from biological replicates using muscle cells from two different human donors. Mean ± SD. ***p < 0.0001; Unpaired t test with Welch’s correction. AU = arbitrary units; GFP = green fluorescent protein; kPA = kilopascals; PLN = phospholamban; SD = standard deviation; shYAP = silencing of yes-associated protein; SMC = smooth muscle cell; YAP = yes-associated protein.

Since both PLN and YAP induced esophageal SMC hypertrophy, we evaluated if there was a positive relationship between the two proteins. Immunofluorescence confirmed the co-expression of YAP and PLN in primary SMCs (Fig. 3A). To understand if YAP was required for mechanotransducive signals that increased PLN on rigid substrate, we silenced YAP expression in SMCs cultured on soft or rigid matrix. YAP silencing obviated PLN induction by rigid substrate (Figs. 3B and 3C). We then evaluated if PLN expression affected YAP when SMCs were cultured on soft substrate which would normally not induce YAP expression and nuclear localization (Fig. 2A). SMCs transduced with PLN and cultured on soft substrates had increased nuclear YAP expression on immunohistochemistry as compared to control (GFP transduced) SMCs (Fig. 3D). Notably, the expression of nuclear YAP in transduced SMCs was assessed following trypsinization, a process that normally removes YAP from the nucleus^28^. The capacity of PLN transduction to retain nuclear YAP despite trypsinization supports a trapping of YAP in the nucleus in response to PLN induction (Fig. 3D).

### PLN over expression enriches for and stabilizes the actin cytoskeleton

**Fig. 4:**
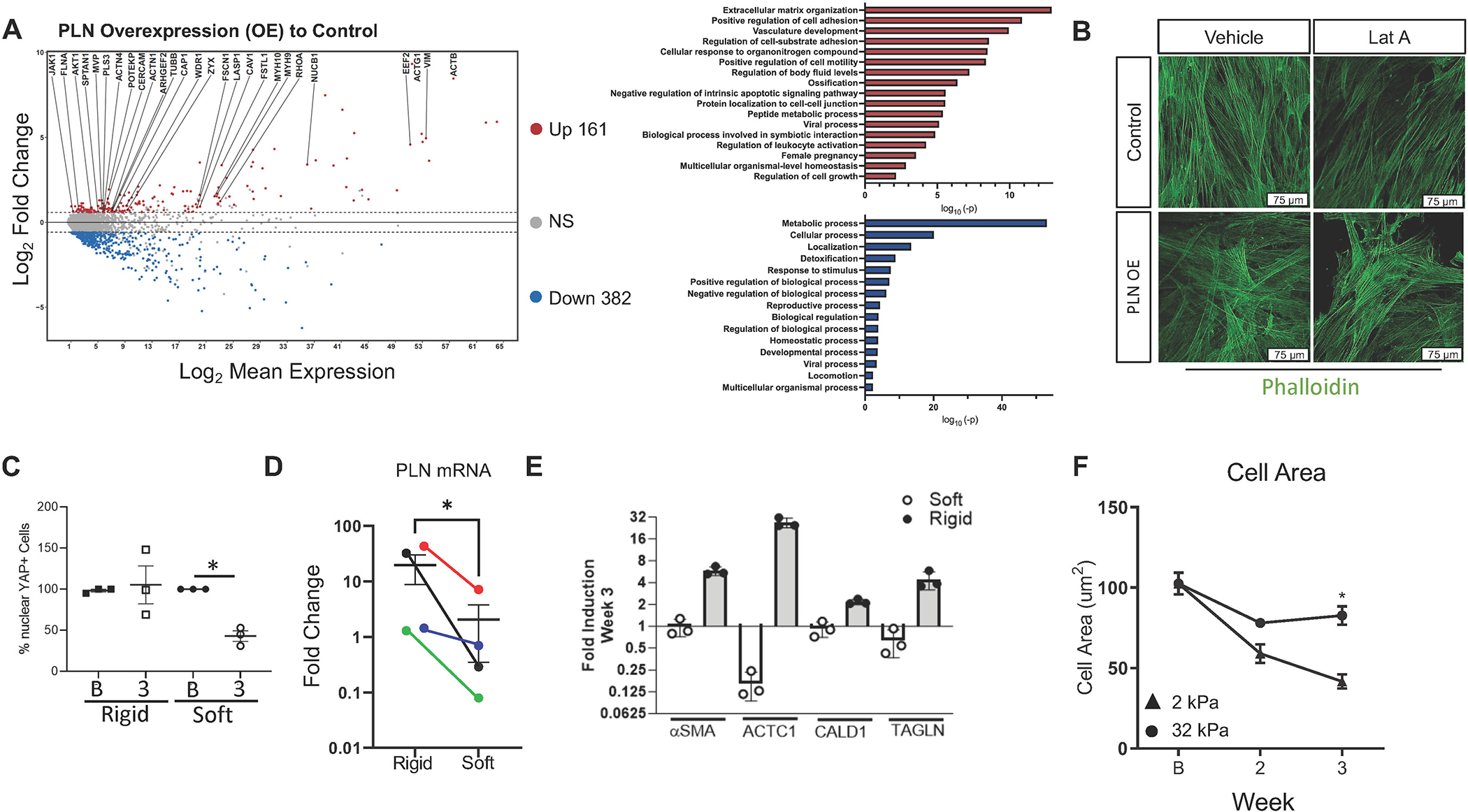
Phospholamban stabilizes the actin cytoskeleton in a reversible manner. (A) Left: Volcano plot of gene microarray of triplicate experiments of esophageal SMCs expressing an inducible PLN transgene treated with vehicle or 2ug/ml of doxycycline for 72 hours. Red and blue dots represent >1.5-fold change of upregulated (*n* = 161) and downregulated (*n* = 382) differentially expressed genes with false discovery rate adjusted *p* < 0.05. Right: GO and Pathway analysis of differentially expressed upregulated (top, red) and downregulated (bottom, blue) genes following doxycycline induction of PLN expression in SMCs. (B) Representative image of phalloidin staining in latrunculin A (0.5uM, 30 minutes) or vehicle treated stable transgenic GFP (control) or PLN-expressing SMCs. <scale bar = 75μm>. (C) Quantitation of nuclear YAP immunostaining (percent of cells that are nuclear YAP+ among total cells) in SMCs cultured on soft or rigid substrate at baseline (B) and after 3 weeks. Dots: different individuals, Lines: Mean ± SEM. *: *p* < .0.05 using analysis of variance with Tukey’s multiple comparisons. (D) Normalized fold change in PLN transcript after 14–21 days (as compared to baseline) in SMCs culture on rigid or soft substrate. Dots = different individuals, lines: Mean ± SEM.*p < 0.05. One-way analysis of variance (Kruskal-Wallis) Dunn’s multiple comparisons test. (E) Representative assay of normalized fold change in mRNA of α-smooth muscle (αSMA) and alpha cardiac muscle 1 (ACTC1) actins, actin-binding proteins caldesmon 1 (CALD1) and transgelin (TAGLN) in SMCs cultured for 21 days (3 weeks) on soft (clear bars) or rigid (solid bars) substrate. Repeated using three different donors. (F) Cell area (μm^2^) on soft (2 kPa) or rigid (32 kPa) substrate at baseline (B), 2, and 3 weeks of culture on soft or rigid substrate. *p < 0.05. Two-way analysis of variance with Sidak’s multiple comparisons test. Dots = mean of 3–5 high power fields ± SEM. Repeated using SMCs from four different donors. αSMA = alpha smooth muscle actin; ACTC1 = actin, alpha, cardiac muscle 1; CALD1 = caldesmon 1; FDR = false discovery rate; GFP = green fluorescent protein; GO = gene ontology; mRNA = messenger ribonucleic acid; PLN = phospholamban; SEM = standard error of the mean; SMC = smooth muscle cell; TAGLN = transgelin; YAP = yes-associated protein.

YAP nuclear localization is influenced by cytoskeleton changes^4^. Cytoskeleton softening during trypsinization of adherent cells is one example of a trigger for translocation of YAP out of the nucleus^20,21^. Given the retention of YAP despite trypsinization in PLN-transduced SMCs (Fig. 3D), we aimed to decipher if PLN could alter cytoskeleton gene expression. Using doxycycline-inducible PLN, we analyzed changes in gene expression. Differential expression analysis revealed that PLN significantly increased cytoskeleton, actin-binding, and extracellular matrix genes such as gut actin-gamma-1, actin beta, and collagens 1A1, 1A2, 3A1, and 6A1(Fig. 4A, red. Supplementary Table 2). Decreased genes included ribosomal proteins (RPS16, 9, 13) and interferon-induced transmembrane proteins (IFITM3, IFITM2) (Fig. 4A, blue, Supplementary Table 2). Predicted gene ontology (GO) enriched pathways included those involved in cell shape including motility and adhesion as well as extracellular matrix organization. Downregulated GO predicted pathways included metabolic processes (Fig. 4A). Functionally, PLN expression stabilized stress fibers in the presence of the actin destabilizer, latrunculin A (Fig. 4B). To decipher if YAP and PLN induction along with SMC hypertrophy were reversible, we cultured cells long-term (21 days) on rigid or soft substrates. Long-term SMC culture on rigid substrate induced chronic nuclear YAP retention (Fig. 4C). In contrast, SMCs cultured long-term on soft substrate decreased nuclear YAP by week 3 (Fig. 4C), demonstrating reversibility on soft substrate. PLN transcript levels also significantly decreased by week 3 in SMCs when cultured on a soft substrate, as compared to rigid substrate (Fig. 4D). Cytoskeleton gene expression was either stable or decreased by week 3 on soft, but not rigid, substrate (Fig. 4E). Lastly, SMC size remained large on rigid substrate but had a reversal in their hypertrophy after long-term culture on soft substrate (Fig. 4F). These data suggest that softening of tissue structure could reverse rigid substrate induced cell hypertrophy by altering PLN expression and YAP colocalization.

In the present study, we show that human donor-derived esophageal SMCs undergo hypertrophy and cytoskeletal changes via PLN-induced YAP sequestration in a mechanotransduction-dependent manner. We demonstrate that PLN overexpression on soft matrix phenocopies the effects of rigid extracelluar matrix (ECM) on nuclear YAP localization and cell hypertrophy. Gene expression analysis of PLN induction revealed enriched pathways for ECM organization, cell adhesion, reduced cell locomotion, and decreased metabolic processes suggesting that a PLN-YAP axis mediates adherent, non-motile, large cells. Furthermore, PLN, nuclear YAP, and cell size were reversed when SMCs were cultured on soft substrate long-term (3 weeks), suggesting that efforts to reduce tissue rigidity could be helpful for normalizing muscle cell dynamics by interrupting a positive feedback loop between PLN and YAP. As an endoplasmic reticulum protein, the effects of PLN on gene transcription are likely to be indirect. It is possible that PLN’s transcriptional effects occur via YAP since PLN overexpression stabilizes the actin cytoskeleton and causes nuclear YAP accumulation. Alternatively, PLN transcriptional effects could occur through altered calcium balance in the cytoplasm and subsequent changes in signal transduction pathways, potentially through direct PLN-YAP interactions, or via altered size of nuclear pores that facilitate protein translocation between the cytoplasm and the nucleus^20^.

Fibrosis with loss of tissue elasticity and muscle hypertrophy are often concurrent features of remodeled hollow organs^1,4,22^. Together, these changes cause reductions in lumen size and eventual loss of organ function. In cardiac and pulmonary disease, this can lead to increased morbidity and mortality^1,4,15^. The lack of reversibility in smooth muscle hypertrophy causes significant disease complications in hollow organ diseases and leaves patients with a dearth of therapies other than resection of strictures, for example in inflammatory bowel disease and mechanical dilation, as in EoE^8^. Here, we show that pediatric EoE patients have increased nuclear YAP-positive cells in the smooth muscle present in esophageal biopsies. Our data suggest that there is a potential positive feedback loop for esophageal lumen stenosis since PLN induces transcription of collagen genes that could induce extracellular matrix scarring with rigidity that could then perpetuate mechanotransduction-induced smooth muscle hypertrophy via PLN and YAP. While each disease of hollow organs has distinct etiologies, common pathogenic pathways may make findings in EoE relevant to diseases such as cardiac hypertrophy in which PLN, and more recently YAP, have been appreciated as major players^4,13,14,18^. These data support that EoE-induced remodeling and fibrostenosis is a complex interplay between tissue rigidity, SMC size, and ensuing collagen deposition.

Our study has limitations. Organ donor-derived primary SMCs are a precious and limited commodity that challenges the replication of the results in large patient groups. However, we have used multiple donor SMCs and found reproducibility in our mechanotransduction system. The necessary use of bioengineered substrates is not an identical replicate of the human esophagus but does provide a mechanism to study the role of soft and rigid substrates on primary SMC function. Since clinical studies also support the idea that rigidity and smooth muscle dysfunction are intricately intertwined in EoE^23^, our studies that reveal a feedback loop between rigidity, hypertrophy, PLN, and YAP are of clinical relevance.

In conclusion, we show that human SMC hypertrophy may be mediated via a PLN-YAP axis. Having demonstrated increased PLN expression in active EoE patients with known tissue hyperplasia^12–14^, we now show increased numbers of YAP+ cells in EoE patients. PLN does not have inherent or direct transcriptional activity, but its role in mediating YAP translocation highlights a PLN-YAP axis as a new mechanism for future studies on how hollow organ disease establishes and progresses. We posit that the PLN-YAP axis may play a critical role in discovering appropriate therapeutic interventions to halt or reverse fibrostenosis in some patients. Future studies to address these mechanisms could be valuable in treating the complications in multiple human disorders of hollow organs.

## METHODS

### Human esophageal smooth muscle cell culture

Studies using human organ donors were not considered human subjects research and were approved under the University of California, San Diego (UCSD) Institutional Review Board (IRB) exempt protocol 130835. Esophageal SMCs were isolated from transplant-grade esophagi obtained through the Arkansas Regional Organ Recovery Agency. Full-thickness esophagus were dissected into mucosal and muscular compartments, and longitudinal, circular, and muscularis mucosa smooth muscle bundles were separated as previously described^12^. Single cells were isolated using collagenase I and cultured in SMC media (SMCM) (ScienCell, Carlsbad, CA, USA) supplemented with 2% fetal bovine serum (FBS), 100 units/ml of penicillin, and 100ug/ml of streptomycin. For short-term experiments, cells were cultured on collagen I coated soft (0.5 or 2kPa) or rigid (32 or 64kPa; Mu Wells, San Diego, CA, USA) 24-plates (25–50,000 cells/well). Collagen I coating was achieved using 1:2000 dilution of bovine I collagen (Advanced Biomatrix, Carlsbad, CA, USA) and incubating at 37°C for 30 minutes. Collagen is then aspirated, wells washed with phosphate-buffered saline (PBS), and cells seeded in complete SMCM overnight and then media switched to basal SMCM (serum- and supplement-free) with 100 units/ml of penicillin and 100 μg/ml of streptomycin for 4 days before collection on day 5. For experiments needing more than 5 days, SMCs were plated on soft or rigid substrate in complete SMCM matching rigidity at each collection timepoint. All studies were conducted using longitudinally oriented passage-matched SMCs at passages 3–6.

### PLN and YAP transduction

Doxycycline-inducible PLN expression constructs and vectors were created by the UCSD Viral Core as previously described^14^. Two short hairpin YAP silences sequences (Addgene, Watertown, Massachusetts, USA ) or scramble were cloned into pLKO.1 lentivirus backbone by the UCSD Viral Core or the authors (E.T.). For transduction, both shYAP lentivirus were transduced simultaneously.

For gene expression microarray studies of PLN overexpression, human primary SMCs (ScienCell, Carlsbad, CA, USA) were stably transfected with doxycycline-inducible PLN virus and cultured in complete SMCM under standard culture conditions on standard tissue cultured plates. Transduced cells were selected using G418 and puromycin (InvivoGen, San Diego,CA, USA). After selection, PLN induction was achieved using 2 μg/ml of doxycycline for 72 hours. Cells were then collected for RNA isolation for microarray analysis. For overexpression of PLN or silencing of YAP, cells were plated on soft or rigid substrate and transduced with virus on day 1. Cells were cultured for additional 4 days for 5 days total and collected for studies. PLN expression and YAP silencing were confirmed using reverse transcription-quantitative polymerase chain reaction (RT-qPCR) and/or immunofluorescence. Transient transduction of PLN or shYAP was completed using attenuated lentiviral constructs and hexadimethrine bromide (Milipore-Sigma,Burlington, Massachusetts).

### Immunofluorescence and immunostaining

For cytospin immunostaining, adherent cells were first collected using trypsin, then cyto-spun and fixed with 4% paraformaldehyde (PFA) before proceeding with immunostaining. For *in situ* immunocyto-histochemistry or -fluorescence, cells were cultured in 4-, 8- or 24-well plates with or without removable glass slides coated with soft and rigid bioengineered substrates, fixed with 4% PFA, and immunostained. Primary antibodies: anti-PLN (Invitrogen, Waltham, Massachusetts, USA; ma3–922), anti-Calnexin (endoplasmic reticulum marker; Abcam, Cambridge, UK; ab13504), and anti-Nuclear Pore Complex (nuclear pore marker; Abcam, Cambridge, UK; ab24609). For quantitation, images were taken at the same gain and magnification and positive cells were counted per high power field. Immunofluorescence images were processed using Fiji (ImageJ build)^24^ matrix autofluorescence was processed using *Subtract Background* function with rolling ball radius at 50 pixels.

For immunohistochemistry of EoE patients’ tissue, archived and paraffin-embedded biopsy specimens were processed as previously described and stained for YAP1 (Abcam, Cambridge, UK;ab56701)^13^. Subjects were enrolled in UCSD/Rady Children’s Hospital San Diego (RCHSD) IRB approved protocols 091485 and 181690 and de-identified clinical data stored in a UCSD Clinical Translational Research Institute Research Electronic Data Capture database. Specimens with adequate muscularis mucosa to evaluate 3–5 high power fields were chosen from patients with severe EoE (defined as non-response to therapy and/or stricture) or from patients with remission EoE, (defined as <15 epithelial eosinophils per high power field at 400x light microscopy). Normal denotes organ donor esophagi.

### Flow cytometry

For evaluation of cell size after PLN overexpression, cells were collected, washed in PBS, and resuspended in flow cytometry buffer (PBS 1X, FBS 2%, and 1% Sodium Azide). For YAP detection, a one-step fixation and permeabilization protocol was used (eBioscience/ThermoFisher, Waltham, Massachusetts, USA; 00–5523). Cells were collected, pelleted, and washed with PBS. Subsequently, cells were stained for viability (Zombie Violet, BioLegend, San Diego, CA, USA; 423113) and washed before proceeding with fixation/permeabilization and antibody staining steps. Cells were stained according to manufacturer’s recommended dilutions: 1:50 rabbit anti-YAP-Alexa Fluor 647 (D8H1X, 38707, Cell Signaling Technology, Danvers, Massachusetts,USA) or 1:1000 rabbit IgG Isotype-Alexa Fluor 647 (DAE1, 3900, Cell Signaling Technology, Danvers, Massachusetts,USA). Flow cytometry was performed on BD Accuri C6 and analyzed using FlowJo v10.

### RNA analysis

Cells were collected from rigid and soft substrates and processed as previously described for RT-PCR^13^. Illumina microarray was analyzed in-house. We first filtered out the probes that did not have gene symbols annotated in the Illumina HT12v4 annotation data (chip IlluminaHuman.v4, San Diego, CA, USA)^25^, resulting in the retention of 33,964 probes. After filtering, the raw probe expression values for the probes in a probe set that defines a gene were consolidated into a single value per sample, yielding 20,910 genes. To remove the unwanted batch effect of the sample processing date, we used the *removeBatchEffect* function of the *limma* package^26^ the batch-effect-corrected gene expression data were then normalized using the *scale* function of R. These normalized gene expression data were then used for differential gene expression analysis. Specifically, the *lmFit* function of the *limma* package^26^ was used to estimate fold changes and standard errors by fitting a linear model for each gene, with empirical Bayes (*eBayes* function) for smoothing the standard errors. After Benjamini-Hochberg false discovery rate correction for multiple testing, genes with adjusted *p* values less than 0.05 and fold change greater than 1.5 were considered as differentially expressed genes. GO and pathway analyses of differential genes were performed using Metascape^27^.

RT-qPCR analysis was completed as previously described and reported as fold change (2^−ΔΔ^)^14^. Relevant primer sequences can be found in the Supplementary Table 1.

### Statistical analysis

All graphs and statistical analysis were completed using GraphPad Prism. The appropriate test was chosen based on the data distribution and the number of variables being assessed. Comparisons were analyzed using unpaired t test with Welch’s comparison. Multiple group analysis was performed using analysis of variance and appropriate multiple comparisons tests and corrections tests as reported in the Figure legends. Two-tailed *p* values <0.05 were considered statistically significant.

## Supplementary Material

Supp Table 1. Primer Sequences

Supplemental Table 2. DEG Up and Down

SuppFigure1_r1

## References

[1] Gokey JJ Active epithelial Hippo signaling in idiopathic pulmonary fibrosis. JCI Insight. 3, e98738 (2018). 29563341 10.1172/jci.insight.98738 PMC5926907

[2] Fox VL, Nurko S, Teitelbaum JE, Badizadegan K & Furuta GT High-resolution EUS in children with eosinophilic “allergic” esophagitis. *Gastrointest*. Endosc. 57, 30–36 (2003). 12518127 10.1067/mge.2003.33

[3] Moosavi S Measuring esophageal compliance using functional lumen imaging probe to assess remodeling in eosinophilic esophagitis. Neurogastroenterol. Motil. 35, e14525 (2023). 36600494 10.1111/nmo.14525 PMC10171050

[4] Panciera T, Azzolin L, Cordenonsi M & Piccolo S Mechanobiology of YAP and TAZ in physiology and disease. Nat. Rev. Mol. Cell Biol. 18, 758–770 (2017). 28951564 10.1038/nrm.2017.87 PMC6192510

[5] Nhu QM & Aceves SS Tissue remodeling in chronic eosinophilic esophageal inflammation: parallels in asthma and therapeutic perspectives. *Front. Med*. **Lausanne**). 4, 128 (2017). 28831387 10.3389/fmed.2017.00128 PMC5549614

[6] Nakamura M & Sadoshima J Mechanisms of physiological and pathological cardiac hypertrophy. Nat. Rev. Cardiol. 15, 387–407 (2018). 29674714 10.1038/s41569-018-0007-y

[7] Hur GY & Broide DH Genes and pathways regulating decline in lung function and airway remodeling in asthma. *Allergy Asthma* *Immunol*. *Res*. 11, 604–621 (2019). 10.4168/aair.2019.11.5.604 PMC6658410 31332973

[8] Dellon ES, Updated international consensus diagnostic criteria for eosinophilic esophagitis: Proceedings of the AGREE Conference. Gastroenterology. 155, 1022–1033.e10 (2018). 30009819 10.1053/j.gastro.2018.07.009 PMC6174113

[9] Alhmoud T, Ghazaleh S, Ghanim M & Redfern RE The risk of esophageal food impaction in eosinophilic esophagitis patients: the role of clinical and socioeconomic factors. Clin. Exp. Gastroenterol. 15, 153–161 (2022). 36132486 10.2147/CEG.S364994 PMC9484774

[10] Warners MJ, Oude Nijhuis RAB, de Wijkerslooth LRH & Smout AJPM & Bredenoord A.J. The natural course of eosinophilic esophagitis and long-term consequences of undiagnosed disease in a large cohort. Am. J. Gastroenterol. 113, 836–844 (2018). 29700481 10.1038/s41395-018-0052-5

[11] Dellon ES & Hirano I Epidemiology and natural history of eosinophilic esophagitis. Gastroenterology. 154, 319–322.e3 (2018). 28774845 10.1053/j.gastro.2017.06.067 PMC5794619

[12] Kurten RC Development and application of a functional human esophageal mucosa explant platform to eosinophilic esophagitis. Sci. Rep. 9, 6206 (2019). 30996235 10.1038/s41598-019-41147-8 PMC6470157

[13] Beppu LY TGF-beta1-induced phospholamban expression alters esophageal smooth muscle cell contraction in patients with eosinophilic esophagitis. *J. Allergy Clin*. *Immunol*. 134, 1100–1107.e4 (2014). 24835503 10.1016/j.jaci.2014.04.004 PMC4231011

[14] Tkachenko E Rigid substrate induces esophageal smooth muscle hypertrophy and eosinophilic esophagitis fibrotic gene expression. J. Allergy Clin. Immunol. 137, 1270–1272.e1 (2016). 26542032 10.1016/j.jaci.2015.09.020 PMC4826849

[15] Vafiadaki E, Glijnis PC, Doevendans PA, Kranias EG & Sanoudou D Phospholamban R14del disease: the past, the present and the future. Front. Cardiovasc. Med. 10, 1162205 (2023). 37144056 10.3389/fcvm.2023.1162205 PMC10151546

[16] Sayedyahossein S, Thines L & Sacks DB Ca(2+) signaling and the Hippo pathway: intersections in cellular regulation. Cell. Signal. 110:110846. 10.1016/j.cellsig.2023.110846 PMC10529277 37549859

[17] Bailey DD Use of hPSC-derived 3D organoids and mouse genetics to define the roles of YAP in the development of the esophagus. Development. 146:dev178855. 10.1242/dev.178855 PMC6918786 31748205

[18] Wang P The alteration of Hippo/YAP signaling in the development of hypertrophic cardiomyopathy. Basic Res. Cardiol. 109, 435 (2014). 25168380 10.1007/s00395-014-0435-8

[19] Laklai H Genotype tunes pancreatic ductal adenocarcinoma tissue tension to induce matricellular fibrosis and tumor progression. Nat. Med. 22, 497–505 (2016). 27089513 10.1038/nm.4082 PMC4860133

[20] Elosegui-Artola A Force triggers YAP nuclear entry by regulating transport across nuclear pores. Cell. 171, 1397–1410.e14 (2017). 29107331 10.1016/j.cell.2017.10.008

[21] Das A, Fischer RS, Pan D & Waterman CM YAP nuclear localization in the absence of cell-cell contact is mediated by a filamentous actin-dependent, myosin II- and phospho-YAP-independent pathway during extracellular matrix mechanosensing. J. Biol. Chem. 291, 6096–6110 (2016). 26757814 10.1074/jbc.M115.708313 PMC4813550

[22] Ou W Increased expression of yes-associated protein/YAP and transcriptional coactivator with PDZ-binding motif/TAZ activates intestinal fibroblasts to promote intestinal obstruction in Crohn’s disease. EBiomedicine. 69:103452. 10.1016/j.ebiom.2021.103452 PMC8243379 34186485

[23] Colizzo JM, Clayton SB & Richter JE Intrabolus pressure on high-resolution manometry distinguishes fibrostenotic and inflammatory phenotypes of eosinophilic esophagitis. Dis. Esophagus. 29, 551–557 (2016). 25913144 10.1111/dote.12360

[24] Schindelin J Fiji: an open-source platform for biological-image analysis. Nat. Methods. 9, 676–682 (2012). 22743772 10.1038/nmeth.2019 PMC3855844

[25] Dunning M, Lynch A, Eldridge M. Illumina HumanHT12v4 annotation data (chip illuminaHumanv4). R package version 1.26.0. Bioconductor. Available at: https://bioconductor.org/packages/release/data/annotation/html/illuminaHumanv4.db.html [Date accessed: XXX].

[26] Ritchie ME limma powers differential expression analyses for RNA-sequencing and microarray studies. Nucleic Acids Res. 43, e47 (2015). 25605792 10.1093/nar/gkv007 PMC4402510

[27] Zhou Y Metascape provides a biologist-oriented resource for the analysis of systems-level datasets. Nat. Commun. 10, 1523 (2019). 30944313 10.1038/s41467-019-09234-6 PMC6447622

[28] Zhao B Cell detachment activates the Hippo pathway via cytoskeleton reorganization to induce anoikis. Genes Dev. 26(1), 54–68 (2012). 10.1101/gad.173435.111. 22215811 PMC3258966

