# Supplementary material for: Mechanotransduction-induced interplay between phospholamban and yes-activated protein induces smooth muscle cell hypertrophy
Source: Mucosal Immunol. Author manuscript; Available in PMC 2024 Jun 24. (PMC11195688; doi:10.1016/j.mucimm.2024.02.007)
Supplement: Supp Table 1. Primer Sequences [file NIHMS1997494-supplement-Supp_Table_1__Primer_Sequences.docx]

| **Supplemental Table 1. qPCR Primer Sequence** | | |
| --- | --- | --- |
| **Gene** | **Forward** | **Reverse** |
| TAGLN1 | CAGAGGAATTGATGGAAACC | ACCCTTACACACACCTTG |
| ACTC1 | GCCCTGGATTTTGAGAATGA | ATGCCAGCAGATTCCATACC |
| CALD1 | CACTAAGGTTTGAGACAGTTCCAGAA | GCGAATTAGCCCTCTACAACTGA |
| ACTA2 (αSMA) | CCGACCGAATGCAGAAGGA | ACAGAGTATTTGCGCTCCGAA |
| PLN | AAACTCCCCAGCTAAACACC | GAACTTCAGAGAAGCATCACGATGATA |
| YAP1 | GCAGGTTGGGAGATGGCAAA | GACGTTCATCTGGGACAGCA |
