# Supplementary material for: Mechanotransduction-induced interplay between phospholamban and yes-activated protein induces smooth muscle cell hypertrophy
Source: Mucosal Immunol. Author manuscript; Available in PMC 2024 Jun 24. (PMC11195688; doi:10.1016/j.mucimm.2024.02.007)
Supplement: Supplemental Table 2. DEG Up and Down [file NIHMS1997494-supplement-Supplemental_Table_2__DEG_Up_and_Down.docx]

| Supplemental Table 2. Differentially expressed up- and down-regulated genes | | | | | | |
| --- | --- | --- | --- | --- | --- | --- |
| Gene | Log_2_Fold Change | Avg exp | t | p-value | p- adj | B |
| ACTG1 | 9.32 | 15.50 | 6.87 | 5.53E-03 | 4.06E-02 | -2.65 |
| ACTB | 8.47 | 17.34 | 11.88 | 1.05E-03 | 1.92E-02 | -0.51 |
| HSPB6 | 7.48 | 11.63 | 22.17 | 1.53E-04 | 7.74E-03 | 1.91 |
| COL6A1 | 6.63 | 12.39 | 10.38 | 1.60E-03 | 2.33E-02 | -1.05 |
| COL1A2 | 5.92 | 19.28 | 47.00 | 1.47E-05 | 3.31E-03 | 4.51 |
| COL3A1 | 5.87 | 18.79 | 26.26 | 9.04E-05 | 6.17E-03 | 2.54 |
| UBC | 5.25 | 12.92 | 19.96 | 2.12E-04 | 8.96E-03 | 1.51 |
| FTL | 5.21 | 15.93 | 66.42 | 5.01E-06 | 2.66E-03 | 5.44 |
| VIM | 4.95 | 16.12 | 20.13 | 2.07E-04 | 8.95E-03 | 1.54 |
| COL1A1 | 4.73 | 15.97 | 8.12 | 3.36E-03 | 3.32E-02 | -2.01 |
| EEF2 | 4.58 | 15.42 | 6.88 | 5.52E-03 | 4.06E-02 | -2.65 |
| GAS6 | 4.31 | 9.37 | 40.20 | 2.40E-05 | 3.74E-03 | 4.02 |
| DCN | 4.27 | 8.41 | 6.13 | 7.75E-03 | 4.48E-02 | -3.09 |
| TGFBI | 3.76 | 12.60 | 26.15 | 9.17E-05 | 6.21E-03 | 2.53 |
| H19 | 3.65 | 11.21 | 10.60 | 1.49E-03 | 2.25E-02 | -0.96 |
| RACK1 | 3.62 | 16.26 | 11.43 | 1.19E-03 | 2.01E-02 | -0.67 |
| LAIR1 | 3.54 | 8.41 | 21.75 | 1.63E-04 | 7.97E-03 | 1.84 |
| PPP2R1A | 3.52 | 6.04 | 7.29 | 4.64E-03 | 3.83E-02 | -2.43 |
| NUCB1 | 3.40 | 10.83 | 10.57 | 1.51E-03 | 2.26E-02 | -0.98 |
| FSTL1 | 3.36 | 7.02 | 4.88 | 1.50E-02 | 4.48E-02 | -3.93 |
| CAVIN1 | 3.34 | 12.00 | 5.77 | 9.27E-03 | 4.48E-02 | -3.32 |
| JUND | 3.28 | 8.34 | 43.69 | 1.85E-05 | 3.47E-03 | 4.29 |
| EEF1A1 | 3.18 | 8.84 | 22.00 | 1.57E-04 | 7.83E-03 | 1.88 |
| POTEF | 2.62 | 7.24 | 67.28 | 4.81E-06 | 2.66E-03 | 5.47 |
| FBLN5 | 2.17 | 4.26 | 10.14 | 1.71E-03 | 2.38E-02 | -1.14 |
| IGFBP4 | 2.14 | 8.16 | 4.40 | 2.00E-02 | 4.49E-02 | -4.30 |
| CYR61 | 2.10 | 3.30 | 14.70 | 5.47E-04 | 1.38E-02 | 0.32 |
| PSAP | 2.07 | 12.57 | 6.04 | 8.11E-03 | 4.48E-02 | -3.15 |
| VASN | 2.07 | 4.91 | 12.06 | 1.01E-03 | 1.88E-02 | -0.46 |
| COL5A1 | 2.06 | 6.87 | 5.95 | 8.46E-03 | 4.48E-02 | -3.20 |
| MTCH1 | 2.00 | 8.97 | 21.51 | 1.68E-04 | 8.07E-03 | 1.80 |
| POTEKP | 1.93 | 1.99 | 4.43 | 1.96E-02 | 4.49E-02 | -4.28 |
| EEF1G | 1.91 | 13.27 | 4.98 | 1.41E-02 | 4.48E-02 | -3.86 |
| CD81 | 1.88 | 14.82 | 4.47 | 1.91E-02 | 4.49E-02 | -4.25 |
| WBP2 | 1.85 | 5.47 | 6.14 | 7.70E-03 | 4.48E-02 | -3.08 |
| LRP10 | 1.82 | 5.30 | 5.61 | 1.00E-02 | 4.48E-02 | -3.42 |
| FBLN1 | 1.82 | 5.64 | 5.52 | 1.05E-02 | 4.48E-02 | -3.48 |
| RPL41 | 1.81 | 8.40 | 11.69 | 1.11E-03 | 1.96E-02 | -0.58 |
| PLTP | 1.79 | 3.27 | 13.27 | 7.51E-04 | 1.59E-02 | -0.08 |
| OLR1 | 1.75 | 2.89 | 15.30 | 4.84E-04 | 1.31E-02 | 0.48 |
| EPN1 | 1.69 | 4.79 | 6.95 | 5.35E-03 | 4.03E-02 | -2.61 |
| MYH9 | 1.67 | 6.82 | 23.26 | 1.32E-04 | 7.28E-03 | 2.09 |
| SEC61A1 | 1.65 | 4.84 | 11.28 | 1.24E-03 | 2.05E-02 | -0.72 |
| COL6A2 | 1.61 | 3.24 | 29.48 | 6.31E-05 | 5.40E-03 | 2.96 |
| LTBR | 1.61 | 2.49 | 34.90 | 3.73E-05 | 4.26E-03 | 3.55 |
| FSCN1 | 1.61 | 6.03 | 11.03 | 1.32E-03 | 2.13E-02 | -0.81 |
| TNFRSF1A | 1.58 | 2.68 | 23.58 | 1.26E-04 | 7.12E-03 | 2.14 |
| SPARC | 1.54 | 9.65 | 10.22 | 1.67E-03 | 2.37E-02 | -1.11 |
| THY1 |  | 6.79 | 6.12 | 7.78E-03 | 4.48E-02 | -3.09 |
| MYH10 | 1.53 | 6.69 | 6.14 | 7.72E-03 | 4.48E-02 | -3.08 |
| RPLP2 | 1.52 | 7.36 | 5.14 | 1.29E-02 | 4.48E-02 | -3.74 |
| ITGB5 | 1.50 | 4.02 | 4.74 | 1.62E-02 | 4.48E-02 | -4.04 |
| COL8A1 | 1.46 | 3.02 | 25.70 | 9.67E-05 | 6.36E-03 | 2.46 |
| TMBIM6 | 1.45 | 6.90 | 29.32 | 6.42E-05 | 5.41E-03 | 2.94 |
| BTBD2 | 1.41 | 3.94 | 6.41 | 6.78E-03 | 4.32E-02 | -2.92 |
| FAM234A | 1.40 | 1.94 | 32.80 | 4.53E-05 | 4.62E-03 | 3.34 |
| COL6A3 | 1.38 | 8.44 | 9.70 | 1.96E-03 | 2.53E-02 | -1.31 |
| MFAP4 | 1.35 | 13.56 | 7.99 | 3.53E-03 | 3.39E-02 | -2.07 |
| OGN | 1.30 | 1.56 | 10.61 | 1.49E-03 | 2.25E-02 | -0.96 |
| PRPF8 | 1.29 | 2.88 | 27.52 | 7.82E-05 | 5.80E-03 | 2.71 |
| EHD2 | 1.29 | 1.66 | 9.53 | 2.07E-03 | 2.60E-02 | -1.38 |
| ANXA2P2 | 1.29 | 12.90 | 22.21 | 1.52E-04 | 7.74E-03 | 1.92 |
| MTMR12 | 1.27 | 5.98 | 8.89 | 2.56E-03 | 2.92E-02 | -1.66 |
| ZDHHC8 | 1.25 | 3.52 | 6.78 | 5.75E-03 | 4.11E-02 | -2.70 |
| RHOA | 1.24 | 6.95 | 7.60 | 4.10E-03 | 3.62E-02 | -2.26 |
| AHCY | 1.23 | 3.35 | 4.07 | 2.48E-02 | 4.58E-02 | -4.58 |
| PTTG1IP | 1.22 | 4.39 | 4.39 | 2.02E-02 | 4.49E-02 | -4.32 |
| GNAI2 | 1.17 | 3.32 | 70.10 | 4.23E-06 | 2.60E-03 | 5.57 |
| EGR1 | 1.15 | 1.96 | 26.37 | 8.93E-05 | 6.17E-03 | 2.56 |
| EMILIN1 | 1.13 | 2.54 | 6.01 | 8.21E-03 | 4.48E-02 | -3.16 |
| C1R | 1.12 | 0.77 | 8.55 | 2.87E-03 | 3.07E-02 | -1.81 |
| PABPC1 | 1.12 | 3.18 | 20.09 | 2.08E-04 | 8.95E-03 | 1.54 |
| LGALS3BP | 1.11 | 1.72 | 15.39 | 4.75E-04 | 1.29E-02 | 0.50 |
| SFRP1 | 1.11 | 6.85 | 4.69 | 1.67E-02 | 4.48E-02 | -4.08 |
| OLFML3 | 1.10 | 3.36 | 26.55 | 8.74E-05 | 6.14E-03 | 2.58 |
| SGSH | 1.08 | 1.99 | 16.05 | 4.17E-04 | 1.22E-02 | 0.67 |
| CDKN1A | 1.06 | 5.87 | 5.39 | 1.13E-02 | 4.48E-02 | -3.57 |
| MAGED1 | 1.06 | 3.26 | 5.94 | 8.49E-03 | 4.48E-02 | -3.21 |
| ZNF594 | 1.04 | 6.86 | 5.02 | 1.38E-02 | 4.48E-02 | -3.83 |
| EIF3L | 1.03 | 7.57 | 4.00 | 2.59E-02 | 4.62E-02 | -4.63 |
| QSOX1 | 1.02 | 3.54 | 21.67 | 1.64E-04 | 8.00E-03 | 1.82 |
| PTBP1 | 0.98 | 4.20 | 27.07 | 8.23E-05 | 5.99E-03 | 2.65 |
| WDR1 | 0.97 | 2.77 | 11.71 | 1.10E-03 | 1.95E-02 | -0.57 |
| CLUAP1 | 0.97 | 2.80 | 60.24 | 6.79E-06 | 2.68E-03 | 5.20 |
| ACTN4 | 0.97 | 1.78 | 8.32 | 3.12E-03 | 3.20E-02 | -1.91 |
| NBPF10 | 0.97 | 0.68 | 6.89 | 5.50E-03 | 4.05E-02 | -2.64 |
| USP5 | 0.96 | 1.51 | 27.89 | 7.50E-05 | 5.74E-03 | 2.76 |
| GRN | 0.96 | 2.63 | 7.82 | 3.76E-03 | 3.48E-02 | -2.16 |
| CYB5R3 | 0.96 | 5.40 | 4.26 | 2.19E-02 | 4.50E-02 | -4.42 |
| SOCS1 | 0.94 | 0.31 | 9.04 | 2.43E-03 | 2.83E-02 | -1.59 |
| CCND2 | 0.94 | 5.72 | 19.35 | 2.34E-04 | 9.35E-03 | 1.39 |
| CAV1 | 0.92 | 6.05 | 6.46 | 6.63E-03 | 4.30E-02 | -2.89 |
| MXD4 | 0.90 | 3.85 | 5.29 | 1.19E-02 | 4.48E-02 | -3.64 |
| VAT1 | 0.90 | 2.42 | 5.24 | 1.22E-02 | 4.48E-02 | -3.68 |
| LASP1 | 0.88 | 5.92 | 4.99 | 1.40E-02 | 4.48E-02 | -3.85 |
| MXRA8 | 0.88 | 0.79 | 30.32 | 5.78E-05 | 5.27E-03 | 3.06 |
| RAB5B | 0.87 | 2.99 | 5.50 | 1.06E-02 | 4.48E-02 | -3.49 |
| RIC8A | 0.87 | 1.74 | 83.84 | 2.42E-06 | 2.17E-03 | 5.94 |
| TXNDC12 | 0.87 | 1.20 | 9.83 | 1.88E-03 | 2.49E-02 | -1.26 |
| SRRM2 | 0.85 | 1.99 | 5.69 | 9.64E-03 | 4.48E-02 | -3.37 |
| ACTN1 | 0.83 | 2.14 | 7.64 | 4.04E-03 | 3.60E-02 | -2.25 |
| LAMB2 | 0.83 | 1.62 | 4.35 | 2.07E-02 | 4.49E-02 | -4.35 |
| TMEM119 | 0.83 | 1.59 | 7.64 | 4.03E-03 | 3.60E-02 | -2.24 |
| FBLN2 | 0.81 | 1.19 | 7.53 | 4.21E-03 | 3.67E-02 | -2.30 |
| UBAP2L | 0.81 | 1.81 | 29.24 | 6.47E-05 | 5.42E-03 | 2.93 |
| RPL3 | 0.81 | 11.01 | 6.76 | 5.81E-03 | 4.12E-02 | -2.72 |
| MVP | 0.80 | 1.67 | 12.81 | 8.37E-04 | 1.70E-02 | -0.22 |
| AKT1 | 0.80 | 1.20 | 39.31 | 2.57E-05 | 3.82E-03 | 3.95 |
| NPIPB13 | 0.80 | 1.73 | 10.09 | 1.74E-03 | 2.39E-02 | -1.16 |
| RRBP1 | 0.79 | 2.08 | 12.41 | 9.23E-04 | 1.81E-02 | -0.34 |
| LZTR1 | 0.79 | 1.89 | 7.29 | 4.64E-03 | 3.83E-02 | -2.42 |
| GARS | 0.78 | 5.53 | 9.31 | 2.22E-03 | 2.72E-02 | -1.47 |
| FLNA | 0.77 | 0.64 | 5.79 | 9.18E-03 | 4.48E-02 | -3.31 |
| SF3A2 | 0.76 | 1.21 | 51.53 | 1.11E-05 | 2.99E-03 | 4.78 |
| CRTAP | 0.76 | 0.59 | 6.43 | 6.74E-03 | 4.32E-02 | -2.91 |
| MAP7D1 | 0.76 | 1.75 | 5.15 | 1.28E-02 | 4.48E-02 | -3.74 |
| TGM2 | 0.76 | 0.89 | 5.26 | 1.21E-02 | 4.48E-02 | -3.66 |
| SHISA5 | 0.76 | 2.85 | 19.84 | 2.16E-04 | 9.02E-03 | 1.49 |
| CTSD | 0.75 | 0.92 | 14.95 | 5.20E-04 | 1.35E-02 | 0.39 |
| FOS | 0.74 | 1.03 | 18.93 | 2.50E-04 | 9.62E-03 | 1.31 |
| ZYX | 0.72 | 2.81 | 8.34 | 3.10E-03 | 3.19E-02 | -1.90 |
| ZMAT3 | 0.72 | 3.74 | 10.17 | 1.70E-03 | 2.38E-02 | -1.13 |
| BSG | 0.72 | 1.98 | 12.87 | 8.25E-04 | 1.69E-02 | -0.20 |
| SLC7A5P2 | 0.72 | 4.44 | 6.35 | 6.99E-03 | 4.36E-02 | -2.96 |
| CHPF | 0.71 | 1.09 | 8.04 | 3.46E-03 | 3.35E-02 | -2.05 |
| AEBP1 | 0.71 | 0.97 | 7.84 | 3.74E-03 | 3.47E-02 | -2.15 |
| SND1 | 0.70 | 2.33 | 7.99 | 3.53E-03 | 3.39E-02 | -2.07 |
| CERCAM | 0.69 | 1.78 | 30.70 | 5.56E-05 | 5.21E-03 | 3.11 |
| MBTPS1 | 0.69 | 1.11 | 19.65 | 2.23E-04 | 9.18E-03 | 1.45 |
| PNPLA6 | 0.69 | 1.33 | 9.15 | 2.34E-03 | 2.78E-02 | -1.54 |
| PABPC4 | 0.69 | 2.91 | 7.02 | 5.18E-03 | 3.99E-02 | -2.57 |
| PCDH18 | 0.69 | 2.70 | 7.28 | 4.66E-03 | 3.83E-02 | -2.43 |
| NCOR2 | 0.69 | 1.06 | 10.84 | 1.40E-03 | 2.18E-02 | -0.87 |
| TUBB | 0.68 | 2.15 | 4.03 | 2.55E-02 | 4.60E-02 | -4.61 |
| ZNF503 | 0.67 | 0.68 | 17.15 | 3.40E-04 | 1.10E-02 | 0.92 |
| CARM1 | 0.67 | 2.47 | 4.22 | 2.24E-02 | 4.51E-02 | -4.45 |
| PYGL | 0.67 | 2.60 | 12.37 | 9.31E-04 | 1.82E-02 | -0.35 |
| CITED2 | 0.67 | 5.39 | 11.54 | 1.15E-03 | 2.00E-02 | -0.63 |
| TTYH3 | 0.65 | 0.40 | 65.77 | 5.16E-06 | 2.66E-03 | 5.42 |
| PDGFRB | 0.65 | 3.98 | 4.30 | 2.12E-02 | 4.49E-02 | -4.38 |
| CLPTM1 | 0.65 | 0.82 | 109.61 | 1.05E-06 | 1.99E-03 | 6.37 |
| ARHGEF2 | 0.65 | 2.10 | 8.47 | 2.96E-03 | 3.11E-02 | -1.84 |
| ADGRA2 | 0.64 | 1.02 | 10.05 | 1.76E-03 | 2.40E-02 | -1.17 |
| JAK1 | 0.64 | 0.39 | 4.11 | 2.42E-02 | 4.56E-02 | -4.55 |
| TPT1 | 0.63 | 5.74 | 5.75 | 9.35E-03 | 4.48E-02 | -3.33 |
| SPTAN1 | 0.63 | 1.51 | 25.32 | 1.01E-04 | 6.47E-03 | 2.41 |
| PLEKHM2 | 0.63 | 2.23 | 3.84 | 2.89E-02 | 4.78E-02 | -4.77 |
| XPNPEP1 | 0.62 | 2.55 | 7.22 | 4.77E-03 | 3.87E-02 | -2.46 |
| POLR2A | 0.62 | 1.94 | 21.48 | 1.69E-04 | 8.07E-03 | 1.79 |
| PLS3 | 0.61 | 1.72 | 35.71 | 3.47E-05 | 4.06E-03 | 3.63 |
| CAP1 | 0.61 | 2.26 | 4.38 | 2.03E-02 | 4.49E-02 | -4.32 |
| PIEZO1 | 0.61 | 1.34 | 9.88 | 1.86E-03 | 2.46E-02 | -1.24 |
| BSDC1 | 0.61 | 1.40 | 18.06 | 2.90E-04 | 1.03E-02 | 1.12 |
| CLSTN1 | 0.61 | 1.20 | 15.92 | 4.28E-04 | 1.23E-02 | 0.63 |
| CSRP1 | 0.61 | 5.55 | 4.95 | 1.44E-02 | 4.48E-02 | -3.88 |
| MAN1B1 | 0.60 | 1.72 | 4.13 | 2.38E-02 | 4.54E-02 | -4.53 |
| PIK3R2 | 0.60 | 3.46 | 8.69 | 2.73E-03 | 3.01E-02 | -1.74 |
| CLPTM1L | 0.59 | 1.17 | 3.73 | 3.12E-02 | 4.95E-02 | -4.87 |
| CCNK | 0.59 | 1.09 | 16.82 | 3.61E-04 | 1.14E-02 | 0.85 |
| ACADVL | 0.59 | 2.62 | 30.23 | 5.84E-05 | 5.27E-03 | 3.05 |
| TIMP3 | 0.59 | 2.58 | 6.25 | 7.34E-03 | 4.43E-02 | -3.02 |
| PMM1 | -0.58 | 0.81 | -34.27 | 3.95E-05 | 4.39E-03 | 3.49 |
| ETFA | -0.58 | 1.57 | -5.35 | 1.15E-02 | 4.48E-02 | -3.60 |
| UNC50 | -0.58 | 1.31 | -5.99 | 8.28E-03 | 4.48E-02 | -3.17 |
| PTGDS | -0.58 | 0.48 | -9.83 | 1.89E-03 | 2.49E-02 | -1.26 |
| NUDT5 | -0.58 | 0.68 | -7.34 | 4.54E-03 | 3.79E-02 | -2.40 |
| HOXA5 | -0.58 | 1.39 | -24.18 | 1.17E-04 | 6.85E-03 | 2.24 |
| POLR3GL | -0.59 | 0.84 | -14.93 | 5.22E-04 | 1.36E-02 | 0.38 |
| WASHC3 | -0.59 | 0.78 | -6.87 | 5.54E-03 | 4.07E-02 | -2.66 |
| RAB10 | -0.59 | 1.87 | -14.64 | 5.54E-04 | 1.40E-02 | 0.31 |
| COX17 | -0.59 | 0.34 | -4.44 | 1.95E-02 | 4.49E-02 | -4.27 |
| TCEAL4 | -0.59 | 0.78 | -7.74 | 3.88E-03 | 3.53E-02 | -2.19 |
| FIS1 | -0.59 | 0.38 | -10.96 | 1.35E-03 | 2.13E-02 | -0.83 |
| PSMB10 | -0.59 | 0.64 | -21.94 | 1.58E-04 | 7.83E-03 | 1.87 |
| RNA18SN5 | -0.60 | 0.33 | -12.87 | 8.24E-04 | 1.69E-02 | -0.20 |
| LSM7 | -0.60 | 0.40 | -4.00 | 2.59E-02 | 4.62E-02 | -4.64 |
| MDH1 | -0.60 | 2.96 | -6.34 | 7.01E-03 | 4.37E-02 | -2.96 |
| EIF1B | -0.60 | 0.97 | -51.67 | 1.10E-05 | 2.99E-03 | 4.79 |
| HNRNPD | -0.60 | 2.29 | -28.39 | 7.10E-05 | 5.65E-03 | 2.83 |
| PSMB2 | -0.60 | 0.55 | -51.32 | 1.12E-05 | 2.99E-03 | 4.77 |
| PITX1 | -0.60 | 0.90 | -25.89 | 9.46E-05 | 6.25E-03 | 2.49 |
| RAB32 | -0.60 | 1.08 | -7.08 | 5.06E-03 | 3.97E-02 | -2.54 |
| RNF181 | -0.60 | 0.32 | -5.94 | 8.50E-03 | 4.48E-02 | -3.21 |
| DECR1 | -0.60 | 1.32 | -67.82 | 4.69E-06 | 2.66E-03 | 5.49 |
| MPV17 | -0.61 | 0.45 | -42.14 | 2.07E-05 | 3.51E-03 | 4.17 |
| NAGK | -0.61 | 0.84 | -13.84 | 6.60E-04 | 1.52E-02 | 0.09 |
| DYNC1LI2 | -0.61 | 3.00 | -4.97 | 1.42E-02 | 4.48E-02 | -3.87 |
| COX7A2L | -0.61 | 2.64 | -5.28 | 1.19E-02 | 4.48E-02 | -3.64 |
| LAGE3 | -0.61 | 0.37 | -4.69 | 1.67E-02 | 4.48E-02 | -4.07 |
| LAMTOR5 | -0.61 | 0.46 | -10.96 | 1.35E-03 | 2.13E-02 | -0.83 |
| DGUOK | -0.61 | 0.57 | -7.85 | 3.72E-03 | 3.46E-02 | -2.14 |
| H2AFJ | -0.61 | 0.38 | -5.89 | 8.71E-03 | 4.48E-02 | -3.24 |
| RPL13AP3 | -0.61 | 2.53 | -16.34 | 3.95E-04 | 1.18E-02 | 0.74 |
| ECH1 | -0.61 | 1.17 | -8.14 | 3.33E-03 | 3.30E-02 | -2.00 |
| EIF4E2 | -0.61 | 0.66 | -13.72 | 6.78E-04 | 1.54E-02 | 0.05 |
| TRAPPC3 | -0.61 | 0.73 | -9.53 | 2.07E-03 | 2.60E-02 | -1.38 |
| UBE2M | -0.61 | 0.89 | -8.28 | 3.16E-03 | 3.22E-02 | -1.93 |
| C19orf53 | -0.61 | 0.40 | -4.61 | 1.76E-02 | 4.48E-02 | -4.14 |
| PCMT1 | -0.61 | 1.46 | -37.18 | 3.06E-05 | 3.91E-03 | 3.77 |
| TRAPPC5 | -0.61 | 0.40 | -5.33 | 1.17E-02 | 4.48E-02 | -3.61 |
| RHOG | -0.62 | 1.29 | -8.37 | 3.07E-03 | 3.18E-02 | -1.89 |
| SNRPF | -0.63 | 0.33 | -5.33 | 1.17E-02 | 4.48E-02 | -3.61 |
| RPS7 | -0.63 | 0.57 | -89.39 | 1.98E-06 | 2.07E-03 | 6.05 |
| YIF1A | -0.63 | 0.84 | -5.00 | 1.39E-02 | 4.48E-02 | -3.84 |
| GLRX | -0.64 | 1.11 | -17.07 | 3.45E-04 | 1.11E-02 | 0.91 |
| CLNS1A | -0.64 | 1.87 | -6.78 | 5.76E-03 | 4.11E-02 | -2.71 |
| AVPI1 | -0.65 | 1.64 | -24.68 | 1.10E-04 | 6.64E-03 | 2.31 |
| KHDRBS1 | -0.65 | 1.45 | -8.51 | 2.92E-03 | 3.10E-02 | -1.83 |
| CENPB | -0.65 | 1.14 | -72.13 | 3.87E-06 | 2.53E-03 | 5.63 |
| BRI3 | -0.65 | 0.79 | -5.28 | 1.20E-02 | 4.48E-02 | -3.65 |
| BTF3 | -0.65 | 3.31 | -42.21 | 2.06E-05 | 3.51E-03 | 4.18 |
| TMEM87A | -0.65 | 1.06 | -15.62 | 4.54E-04 | 1.26E-02 | 0.56 |
| CDK2AP1 | -0.65 | 6.01 | -4.97 | 1.42E-02 | 4.48E-02 | -3.87 |
| UROS | -0.66 | 0.74 | -101.56 | 1.33E-06 | 1.99E-03 | 6.26 |
| COL4A1 | -0.66 | 2.67 | -6.07 | 7.99E-03 | 4.48E-02 | -3.13 |
| HDAC1 | -0.66 | 1.32 | -37.76 | 2.92E-05 | 3.91E-03 | 3.82 |
| ATP5F1D | -0.66 | 0.70 | -6.43 | 6.73E-03 | 4.32E-02 | -2.91 |
| DPYSL2 | -0.66 | 3.62 | -18.96 | 2.49E-04 | 9.61E-03 | 1.31 |
| SSR3 | -0.66 | 0.62 | -16.69 | 3.70E-04 | 1.15E-02 | 0.82 |
| DBI | -0.67 | 0.60 | -5.73 | 9.43E-03 | 4.48E-02 | -3.34 |
| COPRS | -0.67 | 0.57 | -5.11 | 1.32E-02 | 4.48E-02 | -3.77 |
| YIPF3 | -0.67 | 1.30 | -5.23 | 1.23E-02 | 4.48E-02 | -3.68 |
| PGLS | -0.67 | 1.05 | -6.61 | 6.20E-03 | 4.21E-02 | -2.80 |
| AAMDC | -0.67 | 0.38 | -6.66 | 6.07E-03 | 4.17E-02 | -2.77 |
| SSU72 | -0.67 | 1.14 | -106.23 | 1.15E-06 | 1.99E-03 | 6.33 |
| AKR1C3 | -0.68 | 1.34 | -12.31 | 9.45E-04 | 1.83E-02 | -0.37 |
| C1orf123 | -0.68 | 0.73 | -10.35 | 1.61E-03 | 2.34E-02 | -1.06 |
| COX7B | -0.68 | 0.56 | -3.80 | 2.98E-02 | 4.84E-02 | -4.81 |
| CNPY2 | -0.68 | 0.61 | -125.23 | 6.91E-07 | 1.99E-03 | 6.53 |
| SCG5 | -0.68 | 2.09 | -24.66 | 1.10E-04 | 6.64E-03 | 2.31 |
| TRAPPC2L | -0.68 | 0.42 | -5.10 | 1.32E-02 | 4.48E-02 | -3.77 |
| SDHB | -0.68 | 1.12 | -9.26 | 2.26E-03 | 2.75E-02 | -1.49 |
| SWI5 | -0.69 | 0.52 | -6.53 | 6.45E-03 | 4.26E-02 | -2.85 |
| UBE2F | -0.70 | 1.00 | -21.11 | 1.78E-04 | 8.30E-03 | 1.72 |
| NDUFA12 | -0.70 | 0.61 | -70.44 | 4.17E-06 | 2.60E-03 | 5.58 |
| ENY2 | -0.70 | 0.71 | -12.48 | 9.06E-04 | 1.79E-02 | -0.32 |
| VPS29 | -0.70 | 1.20 | -59.56 | 7.04E-06 | 2.68E-03 | 5.17 |
| BASP1 | -0.70 | 5.68 | -15.78 | 4.40E-04 | 1.24E-02 | 0.60 |
| MRPL37 | -0.70 | 1.77 | -4.16 | 2.34E-02 | 4.53E-02 | -4.51 |
| SF3B5 | -0.71 | 0.64 | -4.83 | 1.54E-02 | 4.48E-02 | -3.97 |
| NDUFB7 | -0.71 | 0.50 | -3.99 | 2.62E-02 | 4.63E-02 | -4.65 |
| ACAT1 | -0.71 | 1.50 | -72.62 | 3.79E-06 | 2.53E-03 | 5.64 |
| MRPL36 | -0.71 | 0.59 | -26.00 | 9.33E-05 | 6.24E-03 | 2.51 |
| ATP5F1C | -0.71 | 2.18 | -5.19 | 1.26E-02 | 4.48E-02 | -3.71 |
| RPAIN | -0.71 | 0.78 | -7.10 | 5.01E-03 | 3.95E-02 | -2.53 |
| TOMM5 | -0.71 | 0.62 | -95.84 | 1.59E-06 | 1.99E-03 | 6.17 |
| TCEAL9 | -0.72 | 1.62 | -8.91 | 2.54E-03 | 2.90E-02 | -1.64 |
| PIN1 | -0.72 | 0.77 | -32.51 | 4.65E-05 | 4.68E-03 | 3.31 |
| RNF7 | -0.72 | 0.75 | -12.56 | 8.90E-04 | 1.77E-02 | -0.30 |
| ATP6V1G1 | -0.72 | 1.97 | -19.09 | 2.44E-04 | 9.50E-03 | 1.34 |
| FBN2 | -0.72 | 2.18 | -4.79 | 1.57E-02 | 4.48E-02 | -4.00 |
| EIF3K | -0.72 | 0.63 | -10.67 | 1.47E-03 | 2.23E-02 | -0.94 |
| LSM2 | -0.72 | 0.80 | -11.36 | 1.21E-03 | 2.03E-02 | -0.69 |
| EMC4 | -0.72 | 0.83 | -13.95 | 6.43E-04 | 1.49E-02 | 0.12 |
| PTS | -0.73 | 0.78 | -11.93 | 1.04E-03 | 1.91E-02 | -0.50 |
| RPLP0 | -0.73 | 5.14 | -5.09 | 1.33E-02 | 4.48E-02 | -3.78 |
| BUD31 | -0.73 | 1.59 | -4.08 | 2.47E-02 | 4.58E-02 | -4.57 |
| ERGIC3 | -0.74 | 2.05 | -9.14 | 2.35E-03 | 2.79E-02 | -1.54 |
| SLC25A28 | -0.74 | 1.09 | -5.03 | 1.37E-02 | 4.48E-02 | -3.82 |
| ERH | -0.74 | 0.82 | -138.33 | 5.06E-07 | 1.99E-03 | 6.64 |
| ARL2 | -0.75 | 0.82 | -39.89 | 2.46E-05 | 3.74E-03 | 4.00 |
| RPL23AP7 | -0.75 | 2.26 | -4.36 | 2.05E-02 | 4.49E-02 | -4.34 |
| CAVIN3 | -0.75 | 0.73 | -28.96 | 6.67E-05 | 5.48E-03 | 2.90 |
| PSMG2 | -0.75 | 1.63 | -6.96 | 5.32E-03 | 4.02E-02 | -2.60 |
| THOC7 | -0.75 | 1.54 | -18.82 | 2.55E-04 | 9.74E-03 | 1.29 |
| TMEM147 | -0.76 | 0.77 | -12.65 | 8.71E-04 | 1.75E-02 | -0.27 |
| POLR2H | -0.77 | 1.15 | -24.31 | 1.15E-04 | 6.82E-03 | 2.26 |
| FKBP11 | -0.77 | 0.65 | -13.55 | 7.04E-04 | 1.56E-02 | 0.00 |
| FTH1P3 | -0.77 | 0.76 | -13.45 | 7.20E-04 | 1.57E-02 | -0.03 |
| TMEM179B | -0.77 | 0.73 | -13.37 | 7.34E-04 | 1.59E-02 | -0.05 |
| LSM3 | -0.78 | 0.63 | -5.01 | 1.39E-02 | 4.48E-02 | -3.84 |
| C19orf70 | -0.78 | 0.68 | -5.54 | 1.04E-02 | 4.48E-02 | -3.47 |
| MALSU1 | -0.78 | 1.01 | -7.09 | 5.04E-03 | 3.96E-02 | -2.53 |
| TMBIM4 | -0.78 | 1.65 | -24.19 | 1.17E-04 | 6.85E-03 | 2.24 |
| ATP5MG | -0.79 | 0.96 | -6.05 | 8.07E-03 | 4.48E-02 | -3.14 |
| SDC4 | -0.79 | 1.78 | -4.01 | 2.59E-02 | 4.62E-02 | -4.63 |
| COA3 | -0.79 | 0.72 | -115.75 | 8.83E-07 | 1.99E-03 | 6.44 |
| COMMD1 | -0.80 | 0.84 | -5.02 | 1.38E-02 | 4.48E-02 | -3.83 |
| TAGLN | -0.80 | 3.43 | -7.34 | 4.54E-03 | 3.79E-02 | -2.40 |
| C7orf50 | -0.80 | 1.45 | -9.39 | 2.16E-03 | 2.68E-02 | -1.44 |
| CDK4 | -0.80 | 1.69 | -10.11 | 1.73E-03 | 2.39E-02 | -1.15 |
| ISCU | -0.80 | 1.06 | -59.57 | 7.03E-06 | 2.68E-03 | 5.17 |
| TMEM230 | -0.80 | 2.95 | -4.84 | 1.53E-02 | 4.48E-02 | -3.96 |
| SERPINF1 | -0.80 | 2.08 | -7.30 | 4.62E-03 | 3.83E-02 | -2.42 |
| EMC3 | -0.81 | 1.35 | -18.06 | 2.90E-04 | 1.03E-02 | 1.12 |
| NDUFV2 | -0.81 | 1.28 | -6.07 | 7.96E-03 | 4.48E-02 | -3.12 |
| MT2A | -0.82 | 0.65 | -10.77 | 1.43E-03 | 2.20E-02 | -0.90 |
| SSBP1 | -0.82 | 1.05 | -8.40 | 3.04E-03 | 3.16E-02 | -1.88 |
| HIST1H1C | -0.82 | 0.72 | -9.54 | 2.06E-03 | 2.60E-02 | -1.38 |
| MANF | -0.82 | 1.10 | -11.86 | 1.06E-03 | 1.92E-02 | -0.52 |
| GYPC | -0.82 | 1.13 | -65.86 | 5.14E-06 | 2.66E-03 | 5.42 |
| RTRAF | -0.83 | 1.82 | -31.07 | 5.36E-05 | 5.14E-03 | 3.15 |
| NDUFB5 | -0.83 | 1.26 | -8.77 | 2.66E-03 | 2.97E-02 | -1.71 |
| MYDGF | -0.83 | 0.99 | -7.99 | 3.53E-03 | 3.39E-02 | -2.07 |
| UXT | -0.83 | 0.72 | -5.19 | 1.26E-02 | 4.48E-02 | -3.71 |
| VAMP5 | -0.83 | 0.83 | -18.35 | 2.76E-04 | 1.01E-02 | 1.19 |
| RPL22 | -0.84 | 1.64 | -4.70 | 1.67E-02 | 4.48E-02 | -4.07 |
| UBXN1 | -0.84 | 1.26 | -12.36 | 9.35E-04 | 1.82E-02 | -0.36 |
| CTSB | -0.85 | 2.18 | -7.39 | 4.45E-03 | 3.76E-02 | -2.37 |
| TRAPPC1 | -0.85 | 0.57 | -5.91 | 8.63E-03 | 4.48E-02 | -3.23 |
| CNIH4 | -0.85 | 0.89 | -24.15 | 1.17E-04 | 6.85E-03 | 2.23 |
| BEX3 | -0.85 | 1.31 | -5.40 | 1.12E-02 | 4.48E-02 | -3.56 |
| TST | -0.85 | 1.64 | -6.27 | 7.24E-03 | 4.40E-02 | -3.00 |
| FTH1 | -0.86 | 5.74 | -19.20 | 2.40E-04 | 9.44E-03 | 1.36 |
| ZFAS1 | -0.86 | 1.17 | -5.81 | 9.05E-03 | 4.48E-02 | -3.29 |
| GSTK1 | -0.86 | 1.48 | -10.25 | 1.66E-03 | 2.36E-02 | -1.10 |
| POLR2F | -0.87 | 0.61 | -14.35 | 5.90E-04 | 1.44E-02 | 0.23 |
| OSTC | -0.87 | 4.04 | -4.45 | 1.94E-02 | 4.49E-02 | -4.27 |
| EMD | -0.87 | 1.23 | -36.75 | 3.17E-05 | 3.93E-03 | 3.73 |
| SEM1 | -0.88 | 0.68 | -5.03 | 1.37E-02 | 4.48E-02 | -3.82 |
| SF3B6 | -0.88 | 0.79 | -29.39 | 6.37E-05 | 5.40E-03 | 2.95 |
| LOC729603 | -0.89 | 2.03 | -4.95 | 1.44E-02 | 4.48E-02 | -3.88 |
| WDR83OS | -0.89 | 0.77 | -4.82 | 1.55E-02 | 4.48E-02 | -3.98 |
| CISD1 | -0.89 | 1.13 | -4.84 | 1.53E-02 | 4.48E-02 | -3.96 |
| SS18L2 | -0.90 | 1.15 | -25.63 | 9.76E-05 | 6.38E-03 | 2.45 |
| NDUFB11 | -0.91 | 0.65 | -4.79 | 1.57E-02 | 4.48E-02 | -4.00 |
| SRSF9 | -0.91 | 2.35 | -4.41 | 1.98E-02 | 4.49E-02 | -4.30 |
| NDN | -0.92 | 3.05 | -10.73 | 1.44E-03 | 2.21E-02 | -0.92 |
| NUDC | -0.92 | 1.24 | -5.42 | 1.11E-02 | 4.48E-02 | -3.55 |
| LITAF | -0.92 | 3.18 | -11.73 | 1.10E-03 | 1.95E-02 | -0.57 |
| CPD | -0.92 | 2.69 | -4.78 | 1.58E-02 | 4.48E-02 | -4.01 |
| UFC1 | -0.92 | 1.06 | -145.92 | 4.28E-07 | 1.99E-03 | 6.69 |
| CCND1 | -0.92 | 4.67 | -41.17 | 2.23E-05 | 3.64E-03 | 4.10 |
| TAF10 | -0.92 | 0.89 | -7.98 | 3.54E-03 | 3.39E-02 | -2.08 |
| NDUFA8 | -0.93 | 0.93 | -11.33 | 1.22E-03 | 2.04E-02 | -0.70 |
| CALU | -0.93 | 2.63 | -5.60 | 1.01E-02 | 4.48E-02 | -3.43 |
| COMMD3 | -0.93 | 1.83 | -60.17 | 6.82E-06 | 2.68E-03 | 5.20 |
| RPS6 | -0.93 | 7.80 | -4.90 | 1.48E-02 | 4.48E-02 | -3.92 |
| ORC6 | -0.94 | 3.96 | -4.10 | 2.43E-02 | 4.56E-02 | -4.55 |
| NDUFAB1 | -0.94 | 1.22 | -4.04 | 2.53E-02 | 4.60E-02 | -4.61 |
| EPB41L4A-AS1 | -0.94 | 0.72 | -4.45 | 1.94E-02 | 4.49E-02 | -4.27 |
| POMP | -0.95 | 1.07 | -9.41 | 2.15E-03 | 2.67E-02 | -1.43 |
| URM1 | -0.96 | 1.38 | -9.18 | 2.32E-03 | 2.78E-02 | -1.53 |
| H2AFZ | -0.97 | 1.26 | -13.22 | 7.59E-04 | 1.60E-02 | -0.09 |
| GTF2A2 | -0.97 | 1.15 | -17.43 | 3.23E-04 | 1.07E-02 | 0.99 |
| ZNHIT1 | -0.97 | 0.74 | -6.89 | 5.48E-03 | 4.05E-02 | -2.64 |
| CARHSP1 | -0.97 | 0.98 | -7.56 | 4.17E-03 | 3.65E-02 | -2.29 |
| SPCS1 | -0.97 | 1.98 | -61.25 | 6.45E-06 | 2.68E-03 | 5.24 |
| CIB1 | -0.97 | 1.61 | -29.70 | 6.17E-05 | 5.38E-03 | 2.99 |
| MYL6B | -0.98 | 0.76 | -13.90 | 6.50E-04 | 1.50E-02 | 0.10 |
| ALDH9A1 | -0.99 | 4.97 | -43.86 | 1.83E-05 | 3.47E-03 | 4.30 |
| NOL7 | -0.99 | 1.27 | -10.61 | 1.49E-03 | 2.25E-02 | -0.96 |
| ATP5MC2 | -1.00 | 0.85 | -5.57 | 1.02E-02 | 4.48E-02 | -3.45 |
| ADM | -1.00 | 3.78 | -37.45 | 2.99E-05 | 3.91E-03 | 3.79 |
| PRSS23 | -1.00 | 4.87 | -5.31 | 1.18E-02 | 4.48E-02 | -3.63 |
| CYBA | -1.00 | 0.99 | -12.86 | 8.27E-04 | 1.70E-02 | -0.20 |
| ANAPC13 | -1.01 | 2.79 | -6.32 | 7.09E-03 | 4.39E-02 | -2.97 |
| TOMM6 | -1.01 | 1.25 | -5.01 | 1.39E-02 | 4.48E-02 | -3.84 |
| NEDD8 | -1.01 | 1.36 | -12.85 | 8.29E-04 | 1.70E-02 | -0.21 |
| NDUFS3 | -1.02 | 1.18 | -14.25 | 6.03E-04 | 1.45E-02 | 0.20 |
| RABAC1 | -1.02 | 0.85 | -9.81 | 1.90E-03 | 2.50E-02 | -1.27 |
| COMMD6 | -1.02 | 1.28 | -5.78 | 9.22E-03 | 4.48E-02 | -3.31 |
| KPNB1 | -1.02 | 2.00 | -7.24 | 4.74E-03 | 3.87E-02 | -2.45 |
| NME1 | -1.04 | 0.80 | -4.36 | 2.05E-02 | 4.49E-02 | -4.34 |
| CLIP3 | -1.05 | 4.28 | -6.29 | 7.20E-03 | 4.40E-02 | -2.99 |
| TPM2 | -1.05 | 7.30 | -10.31 | 1.63E-03 | 2.35E-02 | -1.07 |
| TMCO1 | -1.05 | 2.13 | -5.86 | 8.86E-03 | 4.48E-02 | -3.26 |
| NENF | -1.05 | 0.87 | -5.44 | 1.09E-02 | 4.48E-02 | -3.53 |
| EIF3H | -1.06 | 1.89 | -14.14 | 6.17E-04 | 1.46E-02 | 0.17 |
| CUTA | -1.06 | 1.02 | -6.66 | 6.07E-03 | 4.17E-02 | -2.77 |
| EMC7 | -1.06 | 1.64 | -13.58 | 7.00E-04 | 1.56E-02 | 0.01 |
| S100A11 | -1.07 | 0.85 | -8.30 | 3.15E-03 | 3.21E-02 | -1.92 |
| NDUFB3 | -1.07 | 1.38 | -4.20 | 2.27E-02 | 4.51E-02 | -4.47 |
| NDUFS5 | -1.09 | 1.04 | -5.62 | 9.97E-03 | 4.48E-02 | -3.41 |
| CETN2 | -1.10 | 2.08 | -99.02 | 1.44E-06 | 1.99E-03 | 6.22 |
| MRPL33 | -1.10 | 1.02 | -5.20 | 1.25E-02 | 4.48E-02 | -3.70 |
| POLR2G | -1.11 | 1.14 | -25.03 | 1.05E-04 | 6.58E-03 | 2.37 |
| RPS21 | -1.11 | 0.80 | -5.70 | 9.57E-03 | 4.48E-02 | -3.36 |
| NDUFB10 | -1.11 | 1.02 | -4.49 | 1.89E-02 | 4.48E-02 | -4.23 |
| CSNK2B | -1.12 | 1.34 | -38.51 | 2.74E-05 | 3.90E-03 | 3.88 |
| RPL26 | -1.12 | 1.36 | -4.55 | 1.82E-02 | 4.48E-02 | -4.18 |
| DYNLT1 | -1.12 | 1.26 | -5.97 | 8.38E-03 | 4.48E-02 | -3.19 |
| TMED9 | -1.12 | 2.91 | -6.70 | 5.97E-03 | 4.14E-02 | -2.75 |
| MSRB2 | -1.12 | 1.10 | -65.54 | 5.22E-06 | 2.66E-03 | 5.41 |
| SNRPD2 | -1.13 | 1.38 | -5.44 | 1.10E-02 | 4.48E-02 | -3.54 |
| ATP5PD | -1.13 | 1.48 | -5.84 | 8.93E-03 | 4.48E-02 | -3.27 |
| ATP5F1E | -1.13 | 1.06 | -4.23 | 2.24E-02 | 4.51E-02 | -4.45 |
| C6orf48 | -1.13 | 1.20 | -5.84 | 8.95E-03 | 4.48E-02 | -3.27 |
| REXO2 | -1.13 | 3.05 | -6.17 | 7.60E-03 | 4.47E-02 | -3.06 |
| SLC25A6 | -1.13 | 2.85 | -25.24 | 1.02E-04 | 6.49E-03 | 2.40 |
| LSM1 | -1.14 | 1.80 | -5.72 | 9.48E-03 | 4.48E-02 | -3.35 |
| PSMB4 | -1.14 | 1.60 | -14.83 | 5.33E-04 | 1.37E-02 | 0.36 |
| RPL23AP64 | -1.14 | 2.73 | -5.74 | 9.39E-03 | 4.48E-02 | -3.34 |
| RPL6 | -1.15 | 7.50 | -27.06 | 8.24E-05 | 5.99E-03 | 2.65 |
| SELENOM | -1.15 | 1.14 | -8.98 | 2.48E-03 | 2.86E-02 | -1.61 |
| RARRES2 | -1.15 | 1.20 | -6.59 | 6.26E-03 | 4.22E-02 | -2.81 |
| LAMTOR2 | -1.16 | 0.91 | -5.30 | 1.19E-02 | 4.48E-02 | -3.64 |
| PPIA | -1.16 | 1.97 | -51.19 | 1.13E-05 | 2.99E-03 | 4.76 |
| TXNDC17 | -1.17 | 1.19 | -5.84 | 8.92E-03 | 4.48E-02 | -3.27 |
| MRPS6 | -1.17 | 1.77 | -21.02 | 1.81E-04 | 8.35E-03 | 1.71 |
| PHPT1 | -1.19 | 1.30 | -3.84 | 2.90E-02 | 4.79E-02 | -4.78 |
| MYL12B | -1.20 | 2.54 | -8.13 | 3.34E-03 | 3.31E-02 | -2.00 |
| PSMA5 | -1.20 | 2.29 | -104.45 | 1.22E-06 | 1.99E-03 | 6.30 |
| EIF3G | -1.20 | 1.71 | -78.73 | 2.94E-06 | 2.37E-03 | 5.81 |
| SEC11A | -1.20 | 2.32 | -62.55 | 6.04E-06 | 2.68E-03 | 5.30 |
| PSMB3 | -1.20 | 1.29 | -11.86 | 1.06E-03 | 1.92E-02 | -0.52 |
| MRPL51 | -1.20 | 1.11 | -9.58 | 2.04E-03 | 2.59E-02 | -1.36 |
| ZNF674 | -1.21 | 8.01 | -3.79 | 3.00E-02 | 4.85E-02 | -4.82 |
| RPL10 | -1.22 | 5.49 | -4.85 | 1.52E-02 | 4.48E-02 | -3.95 |
| HIST1H2BK | -1.22 | 1.87 | -37.05 | 3.10E-05 | 3.91E-03 | 3.75 |
| EDF1 | -1.22 | 1.34 | -5.33 | 1.17E-02 | 4.48E-02 | -3.61 |
| ATP6V1F | -1.22 | 1.24 | -6.50 | 6.51E-03 | 4.28E-02 | -2.86 |
| ATP5PF | -1.23 | 1.29 | -8.80 | 2.64E-03 | 2.97E-02 | -1.70 |
| GLRX5 | -1.23 | 2.19 | -6.97 | 5.31E-03 | 4.02E-02 | -2.60 |
| SNHG8 | -1.27 | 1.45 | -6.09 | 7.91E-03 | 4.48E-02 | -3.11 |
| COX6A1 | -1.27 | 0.86 | -5.29 | 1.19E-02 | 4.48E-02 | -3.64 |
| MT1A | -1.27 | 0.90 | -7.02 | 5.20E-03 | 3.99E-02 | -2.57 |
| HDDC2 | -1.28 | 1.72 | -5.51 | 1.06E-02 | 4.48E-02 | -3.49 |
| ZNF428 | -1.29 | 1.85 | -10.60 | 1.49E-03 | 2.25E-02 | -0.96 |
| GAPDH | -1.29 | 3.82 | -63.37 | 5.80E-06 | 2.68E-03 | 5.33 |
| DDT | -1.29 | 1.07 | -6.13 | 7.75E-03 | 4.48E-02 | -3.09 |
| RBX1 | -1.30 | 1.17 | -4.58 | 1.79E-02 | 4.48E-02 | -4.17 |
| UQCRH | -1.30 | 1.56 | -6.06 | 8.03E-03 | 4.48E-02 | -3.13 |
| RPSA | -1.31 | 1.56 | -45.63 | 1.62E-05 | 3.42E-03 | 4.42 |
| RPL27A | -1.31 | 14.14 | -3.91 | 2.76E-02 | 4.70E-02 | -4.72 |
| ATRAID | -1.31 | 2.43 | -33.92 | 4.08E-05 | 4.47E-03 | 3.46 |
| RHOC | -1.31 | 5.27 | -3.93 | 2.72E-02 | 4.68E-02 | -4.70 |
| RPL21 | -1.32 | 1.12 | -13.49 | 7.13E-04 | 1.57E-02 | -0.01 |
| EEF1B2 | -1.33 | 2.91 | -18.71 | 2.60E-04 | 9.83E-03 | 1.26 |
| RPL15 | -1.33 | 5.16 | -4.81 | 1.55E-02 | 4.48E-02 | -3.98 |
| RTL8C | -1.34 | 3.40 | -9.54 | 2.06E-03 | 2.60E-02 | -1.38 |
| C12orf57 | -1.35 | 1.25 | -5.31 | 1.17E-02 | 4.48E-02 | -3.62 |
| SH3BGRL3 | -1.36 | 1.53 | -4.48 | 1.90E-02 | 4.48E-02 | -4.24 |
| CHCHD2 | -1.37 | 2.11 | -30.68 | 5.57E-05 | 5.21E-03 | 3.10 |
| NDUFS4 | -1.38 | 1.39 | -15.30 | 4.84E-04 | 1.31E-02 | 0.48 |
| NDUFA11 | -1.39 | 1.07 | -9.49 | 2.10E-03 | 2.62E-02 | -1.40 |
| POLE4 | -1.39 | 1.49 | -16.43 | 3.88E-04 | 1.17E-02 | 0.76 |
| HEBP1 | -1.40 | 3.13 | -6.84 | 5.62E-03 | 4.08E-02 | -2.67 |
| PSMB5 | -1.41 | 2.00 | -23.68 | 1.25E-04 | 7.06E-03 | 2.16 |
| NDUFB2 | -1.46 | 1.32 | -4.79 | 1.57E-02 | 4.48E-02 | -4.00 |
| HIGD2A | -1.47 | 1.24 | -6.12 | 7.79E-03 | 4.48E-02 | -3.10 |
| GTF2H5 | -1.48 | 1.58 | -4.82 | 1.55E-02 | 4.48E-02 | -3.98 |
| NDUFB8 | -1.49 | 1.52 | -9.58 | 2.04E-03 | 2.59E-02 | -1.36 |
| SAT1 | -1.52 | 3.24 | -4.66 | 1.70E-02 | 4.48E-02 | -4.10 |
| SLIRP | -1.53 | 1.90 | -4.05 | 2.50E-02 | 4.59E-02 | -4.59 |
| CAMLG | -1.53 | 3.94 | -10.54 | 1.52E-03 | 2.27E-02 | -0.99 |
| RPS3 | -1.54 | 4.01 | -7.38 | 4.47E-03 | 3.77E-02 | -2.38 |
| UCHL1 | -1.54 | 4.22 | -35.99 | 3.39E-05 | 3.99E-03 | 3.66 |
| NDUFA1 | -1.55 | 1.77 | -4.92 | 1.46E-02 | 4.48E-02 | -3.91 |
| TRMT112 | -1.56 | 2.00 | -28.33 | 7.14E-05 | 5.65E-03 | 2.82 |
| MDH2 | -1.58 | 6.03 | -6.74 | 5.85E-03 | 4.13E-02 | -2.72 |
| AURKAIP1 | -1.59 | 1.41 | -11.18 | 1.27E-03 | 2.07E-02 | -0.75 |
| PRDX4 | -1.59 | 3.71 | -11.95 | 1.04E-03 | 1.90E-02 | -0.49 |
| RPS8 | -1.60 | 4.48 | -9.11 | 2.37E-03 | 2.80E-02 | -1.56 |
| B2M | -1.61 | 7.69 | -27.87 | 7.52E-05 | 5.74E-03 | 2.76 |
| CFL1 | -1.61 | 3.59 | -18.00 | 2.92E-04 | 1.03E-02 | 1.11 |
| RPL23A | -1.62 | 3.31 | -5.34 | 1.16E-02 | 4.48E-02 | -3.60 |
| VKORC1 | -1.62 | 1.89 | -5.43 | 1.10E-02 | 4.48E-02 | -3.54 |
| RPS20 | -1.62 | 4.21 | -3.90 | 2.79E-02 | 4.72E-02 | -4.73 |
| NBDY | -1.66 | 2.15 | -5.62 | 9.97E-03 | 4.48E-02 | -3.41 |
| PSMB7 | -1.69 | 1.98 | -172.46 | 2.54E-07 | 1.99E-03 | 6.82 |
| TSPO | -1.70 | 2.47 | -5.59 | 1.01E-02 | 4.48E-02 | -3.43 |
| RPS27L | -1.71 | 2.82 | -6.45 | 6.66E-03 | 4.31E-02 | -2.89 |
| IGFBP7 | -1.73 | 2.88 | -16.25 | 4.02E-04 | 1.19E-02 | 0.71 |
| NOP10 | -1.74 | 1.77 | -5.75 | 9.36E-03 | 4.48E-02 | -3.33 |
| PSMB6 | -1.77 | 2.05 | -26.65 | 8.64E-05 | 6.09E-03 | 2.60 |
| RPL14 | -1.79 | 2.30 | -5.88 | 8.76E-03 | 4.48E-02 | -3.25 |
| SEC61G | -1.81 | 2.40 | -6.19 | 7.55E-03 | 4.46E-02 | -3.05 |
| NDUFS8 | -1.81 | 1.61 | -17.69 | 3.09E-04 | 1.05E-02 | 1.04 |
| FABP4 | -1.82 | 1.44 | -17.05 | 3.46E-04 | 1.11E-02 | 0.90 |
| RPL21P28 | -1.82 | 1.82 | -9.14 | 2.35E-03 | 2.79E-02 | -1.55 |
| GUK1 | -1.82 | 2.75 | -8.87 | 2.57E-03 | 2.93E-02 | -1.66 |
| SRP14 | -1.83 | 2.77 | -100.99 | 1.35E-06 | 1.99E-03 | 6.25 |
| RPL12 | -1.83 | 3.26 | -12.19 | 9.74E-04 | 1.85E-02 | -0.41 |
| DYNLL1 | -1.84 | 2.48 | -6.12 | 7.80E-03 | 4.48E-02 | -3.10 |
| ATP6V0E1 | -1.84 | 3.07 | -6.83 | 5.64E-03 | 4.09E-02 | -2.68 |
| HSPB1 | -1.85 | 4.65 | -6.70 | 5.96E-03 | 4.14E-02 | -2.75 |
| TMEM14C | -1.85 | 2.70 | -23.70 | 1.24E-04 | 7.06E-03 | 2.16 |
| SEC61B | -1.87 | 2.73 | -4.17 | 2.31E-02 | 4.52E-02 | -4.49 |
| ATP5PO | -1.87 | 2.90 | -130.07 | 6.14E-07 | 1.99E-03 | 6.58 |
| COX5A | -1.88 | 2.60 | -9.23 | 2.28E-03 | 2.76E-02 | -1.51 |
| COX7A2 | -1.88 | 2.40 | -5.18 | 1.26E-02 | 4.48E-02 | -3.72 |
| UQCRBP1 | -1.89 | 2.83 | -5.54 | 1.04E-02 | 4.48E-02 | -3.47 |
| RPL27 | -1.91 | 3.78 | -5.48 | 1.07E-02 | 4.48E-02 | -3.51 |
| CSTB | -1.92 | 2.76 | -11.64 | 1.12E-03 | 1.97E-02 | -0.60 |
| PRDX5 | -1.92 | 2.97 | -4.47 | 1.91E-02 | 4.49E-02 | -4.25 |
| PARK7 | -1.93 | 2.91 | -16.73 | 3.67E-04 | 1.14E-02 | 0.83 |
| RPL9 | -1.95 | 3.93 | -27.61 | 7.74E-05 | 5.79E-03 | 2.73 |
| JTB | -1.96 | 5.13 | -13.85 | 6.57E-04 | 1.51E-02 | 0.09 |
| TBCA | -1.97 | 2.15 | -9.27 | 2.25E-03 | 2.75E-02 | -1.49 |
| AP2S1 | -1.98 | 4.56 | -8.67 | 2.75E-03 | 3.01E-02 | -1.75 |
| RPL13A | -2.00 | 8.95 | -10.54 | 1.52E-03 | 2.27E-02 | -0.99 |
| COX8A | -2.03 | 1.93 | -4.16 | 2.34E-02 | 4.53E-02 | -4.51 |
| UBA52 | -2.05 | 6.08 | -4.87 | 1.51E-02 | 4.48E-02 | -3.94 |
| RPL13AP6 | -2.05 | 2.61 | -5.68 | 9.68E-03 | 4.48E-02 | -3.38 |
| COX5B | -2.07 | 2.24 | -5.82 | 9.00E-03 | 4.48E-02 | -3.28 |
| RRAS | -2.11 | 2.19 | -9.80 | 1.90E-03 | 2.50E-02 | -1.27 |
| COX6B1 | -2.12 | 2.72 | -4.46 | 1.92E-02 | 4.49E-02 | -4.26 |
| RPL29 | -2.13 | 3.52 | -5.80 | 9.10E-03 | 4.48E-02 | -3.30 |
| GSTO1 | -2.13 | 2.54 | -12.22 | 9.68E-04 | 1.85E-02 | -0.40 |
| RPS26P11 | -2.18 | 3.74 | -4.99 | 1.40E-02 | 4.48E-02 | -3.85 |
| NACA | -2.18 | 8.92 | -7.66 | 4.00E-03 | 3.58E-02 | -2.23 |
| MGST3 | -2.19 | 2.67 | -5.68 | 9.68E-03 | 4.48E-02 | -3.38 |
| RPL10A | -2.21 | 3.79 | -8.99 | 2.47E-03 | 2.85E-02 | -1.61 |
| RPS17 | -2.24 | 6.06 | -5.76 | 9.30E-03 | 4.48E-02 | -3.32 |
| UQCRHL | -2.26 | 3.70 | -4.30 | 2.13E-02 | 4.49E-02 | -4.38 |
| PSMB1 | -2.26 | 4.06 | -10.53 | 1.52E-03 | 2.27E-02 | -0.99 |
| COX4I1 | -2.29 | 4.79 | -7.19 | 4.84E-03 | 3.89E-02 | -2.48 |
| RPS10 | -2.35 | 10.77 | -4.93 | 1.45E-02 | 4.48E-02 | -3.89 |
| PRDX1 | -2.36 | 4.21 | -21.23 | 1.75E-04 | 8.24E-03 | 1.75 |
| HINT1 | -2.40 | 3.97 | -9.95 | 1.81E-03 | 2.44E-02 | -1.21 |
| RPL8 | -2.43 | 3.97 | -75.53 | 3.35E-06 | 2.42E-03 | 5.73 |
| UBB | -2.58 | 6.04 | -14.05 | 6.30E-04 | 1.48E-02 | 0.15 |
| DAD1 | -2.60 | 3.70 | -77.21 | 3.13E-06 | 2.40E-03 | 5.77 |
| GPX4 | -2.63 | 6.81 | -7.98 | 3.53E-03 | 3.39E-02 | -2.07 |
| PLIN3 | -2.63 | 4.10 | -18.08 | 2.88E-04 | 1.03E-02 | 1.13 |
| TXN | -2.67 | 2.86 | -22.92 | 1.38E-04 | 7.31E-03 | 2.04 |
| CST3 | -2.69 | 9.97 | -30.15 | 5.89E-05 | 5.29E-03 | 3.04 |
| MIF | -2.71 | 3.01 | -4.74 | 1.63E-02 | 4.48E-02 | -4.04 |
| SSR4 | -2.74 | 2.63 | -5.43 | 1.10E-02 | 4.48E-02 | -3.54 |
| RPL24 | -2.74 | 5.23 | -4.11 | 2.41E-02 | 4.55E-02 | -4.54 |
| RPL18A | -2.77 | 6.67 | -11.51 | 1.16E-03 | 2.00E-02 | -0.64 |
| RPL19 | -2.78 | 7.86 | -9.51 | 2.08E-03 | 2.61E-02 | -1.39 |
| S100A6 | -2.81 | 4.99 | -6.37 | 6.92E-03 | 4.35E-02 | -2.94 |
| OST4 | -2.82 | 3.93 | -4.06 | 2.49E-02 | 4.59E-02 | -4.59 |
| RPS2P32 | -2.83 | 3.18 | -43.14 | 1.93E-05 | 3.48E-03 | 4.25 |
| COX6C | -2.84 | 3.65 | -4.34 | 2.07E-02 | 4.49E-02 | -4.35 |
| NPC2 | -2.84 | 6.28 | -22.85 | 1.39E-04 | 7.31E-03 | 2.02 |
| SOD1 | -2.98 | 4.53 | -11.10 | 1.30E-03 | 2.10E-02 | -0.78 |
| LRRC75A-AS1 | -3.02 | 6.95 | -4.04 | 2.53E-02 | 4.60E-02 | -4.60 |
| RPL23AP87 | -3.21 | 3.45 | -4.13 | 2.38E-02 | 4.54E-02 | -4.53 |
| GSTP1 | -3.23 | 4.67 | -12.10 | 9.97E-04 | 1.87E-02 | -0.44 |
| RPL36AL | -3.27 | 4.12 | -7.05 | 5.13E-03 | 3.99E-02 | -2.56 |
| RPL18 | -3.32 | 6.01 | -6.26 | 7.30E-03 | 4.42E-02 | -3.01 |
| EMP3 | -3.32 | 4.74 | -5.15 | 1.28E-02 | 4.48E-02 | -3.74 |
| IFITM3 | -3.35 | 3.36 | -6.59 | 6.25E-03 | 4.22E-02 | -2.81 |
| PFN1 | -3.40 | 5.73 | -56.51 | 8.29E-06 | 2.76E-03 | 5.03 |
| RPS9 | -3.40 | 4.03 | -8.06 | 3.44E-03 | 3.34E-02 | -2.04 |
| MGP | -3.50 | 4.73 | -3.89 | 2.80E-02 | 4.73E-02 | -4.74 |
| RPS11 | -3.65 | 8.34 | -4.45 | 1.94E-02 | 4.49E-02 | -4.27 |
| RPS13 | -3.65 | 4.94 | -9.35 | 2.19E-03 | 2.70E-02 | -1.46 |
| RPL11 | -3.65 | 11.90 | -8.03 | 3.47E-03 | 3.36E-02 | -2.05 |
| RPL30 | -3.71 | 8.36 | -62.56 | 6.04E-06 | 2.68E-03 | 5.30 |
| RPS14 | -3.82 | 5.07 | -7.26 | 4.69E-03 | 3.84E-02 | -2.44 |
| RPS15 | -3.88 | 6.24 | -4.17 | 2.31E-02 | 4.52E-02 | -4.49 |
| RPS5 | -3.99 | 8.52 | -6.95 | 5.35E-03 | 4.03E-02 | -2.61 |
| PFDN5 | -4.00 | 5.98 | -6.27 | 7.24E-03 | 4.41E-02 | -3.00 |
| RPL35A | -4.54 | 8.90 | -5.13 | 1.30E-02 | 4.48E-02 | -3.75 |
| FAU | -4.61 | 4.62 | -9.25 | 2.26E-03 | 2.75E-02 | -1.50 |
| RPS16 | -4.72 | 9.88 | -5.93 | 8.52E-03 | 4.48E-02 | -3.21 |
| MYL6 | -5.05 | 8.27 | -4.45 | 1.94E-02 | 4.49E-02 | -4.27 |
| IFITM2 | -5.23 | 6.14 | -6.38 | 6.89E-03 | 4.35E-02 | -2.94 |
| LGALS1 | -6.22 | 10.60 | -4.69 | 1.67E-02 | 4.48E-02 | -4.08 |
