# Supplementary figures and images for: Mechanotransduction-induced interplay between phospholamban and yes-activated protein induces smooth muscle cell hypertrophy
Source: Mucosal Immunol. Author manuscript; Available in PMC 2024 Jun 24. (PMC11195688; doi:10.1016/j.mucimm.2024.02.007)

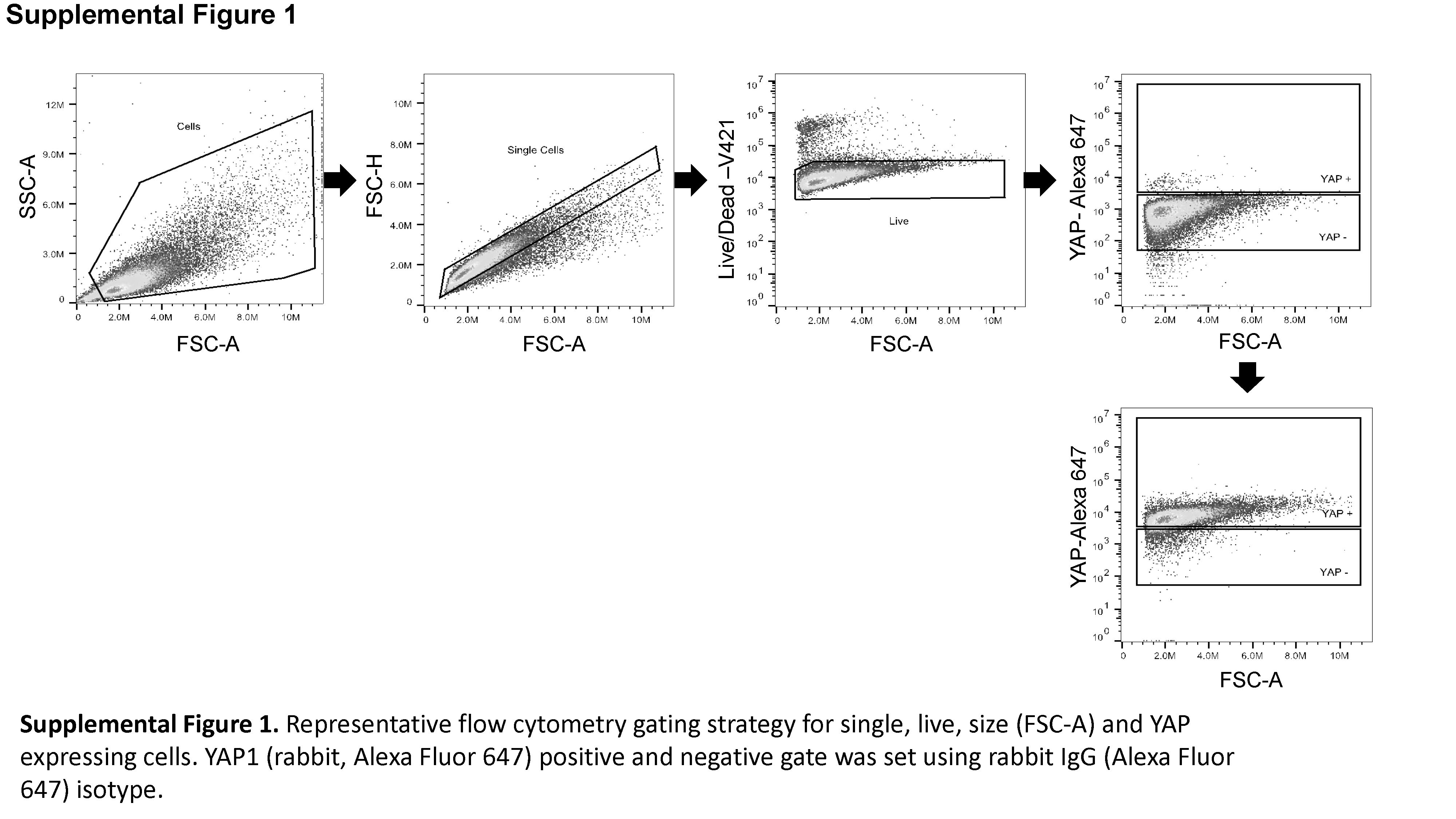

Supplement: SuppFigure1_r1 [file NIHMS1997494-supplement-SuppFigure1_r1.jpg]
